# Stage-Dependent and Locus-Specific Role of Histone Demethylase Jumonji D3 (JMJD3) in the Embryonic Stages of Lung Development

**DOI:** 10.1371/journal.pgen.1004524

**Published:** 2014-07-31

**Authors:** Qingtian Li, Helen Y. Wang, Iouri Chepelev, Qingyuan Zhu, Gang Wei, Keji Zhao, Rong-Fu Wang

**Affiliations:** 1 Center for Inflammation and Epigenetics, Houston Methodist Research Institute, Houston, Texas, United States of America; 2 Center for Autoimmune Genomics and Etiology, Cincinnati Children's Hospital Medical Center, Cincinnati, Ohio, United States of America; 3 CAS-MPG Partner Institute for Computational Biology, Shanghai Institutes of Biological Sciences, Chinese Academy of Sciences, Shanghai, China; 4 Laboratory of Molecular Immunology, National Heart, Lung, and Blood Institute, NIH, Bethesda, Maryland, United States of America; Massachusetts General Hospital, Howard Hughes Medical Institute, United States of America

## Abstract

Histone demethylases have emerged as important players in developmental processes. Jumonji domain containing-3 (Jmjd3) has been identified as a key histone demethylase that plays a critical role in the regulation of gene expression; however, the *in vivo* function of Jmjd3 in embryonic development remains largely unknown. To this end, we generated *Jmjd3* global and conditional knockout mice. Global deletion of *Jmjd3* induces perinatal lethality associated with defective lung development. Tissue and stage-specific deletion revealed that Jmjd3 is dispensable in the later stage of embryonic lung development. *Jmjd3* ablation downregulates the expression of genes critical for lung development and function, including *AQP-5* and *SP-B*. Jmjd3-mediated alterations in gene expression are associated with locus-specific changes in the methylation status of H3K27 and H3K4. Furthermore, Jmjd3 is recruited to the *SP-B* promoter through interactions with the transcription factor Nkx2.1 and the epigenetic protein Brg1. Taken together, these findings demonstrate that *Jmjd3* plays a stage-dependent and locus-specific role in the mouse lung development. Our study provides molecular insights into the mechanisms by which Jmjd3 regulates target gene expression in the embryonic stages of lung development.

## Author Summary

A chromosome in the eukaryotic nucleus is an organized package of DNA coiled around histone proteins. DNA contains genes and other nucleotide sequences as well as histone proteins including H1, H2A, H2B, H3, and H4. Gene expression is dynamically regulated by DNA and histone modifications, such as methylation and demethylation. The protein Jumonji domain containing-3 (Jmjd3) is a critical demethylase that regulates gene expression. Here, we found that Jmjd3 plays an important role in the regulation of mouse lung development. Global *Jmjd3* deletion results in perinatal lethality that is associated with respiratory failure caused by defective lung development. Tissue and stage-specific deletion show that Jmjd3 is dispensable for mouse lung development in the later stages (after E9.5). *Jmjd3* deficiency downregulates the expression of genes critical for lung development through interactions with specific transcription factors and epigenetic protein complexes. Our findings provide new insights into the role and molecular mechanism of action of Jmjd3 in embryonic lung development.

## Introduction

Gene expression is epigenetically regulated through DNA methylation as well as covalent chromatin modifications such as acetylation, phosphorylation, ubiquitination, sumoylation, and methylation of histones. Histone methylation state is dynamically regulated by histone methyltransferases and demethylases [1]–[5]. The trimethylation of histone 3 (H3K4) at lysine 4 is usually associated with the activation of gene expression, whereas trimethylation of histone 3 at lysine 27 (H3K27) is associated with the repression of gene expression [1]–[5]. The polycomb repressive complex, which contains the H3K27 methyltransferase Ezh2 [5], [6], dimethylates and trimethylates H3K27 (H3K27me2/3). Recently, the H3K27 demethylase Jumonji domain containing-3 (Jmjd3; KDM6B) was found to catalyze the demethylation of H3K27me2/3 *in vitro* [7]–[11].

Despite the identification of Jmjd3 as a key H3K27 demethylase, little is known regarding its *in vivo* function. Jmjd3 expression is induced by vitamin D and proinflammatory stimuli in macrophages and is required for the expression of *INK4A*-*ARF*, *Nodal*, and *Irf4* in fibroblasts, mouse embryonic stem cells (ESCs), and macrophages, respectively [12]–[16]. Recently, we showed that Jmjd3 plays a vital role in induced pluripotent stem cell reprogramming by regulating INK4a/Arf expression and PHF20 degradation [17]. Several studies using *Jmjd3* knockout (KO) mice have demonstrated the importance of Jmjd3 in differentiation and development *in vivo*. Jmjd3 has been shown to play a crucial role in the regulation of macrophage development and differentiation [15] and mesoderm differentiation and cardiovascular lineage commitment in mouse ESCs [18]. Furthermore, the embryonic and postnatal lethality of *Jmjd3* deletion in mice indicate the critical requirement for Jmjd3 during development [15], [18], [19]. However, the role and mechanism of action of Jmjd3 in differentiation and developmental processes remain largely unknown.

Lung development is a complex process that requires the participation of many transcription factors and developmentally regulated genes at several different stages. This complex process begins with the formation of airways from embryonic lung buds that originate from the foregut endoderm and branch into the millions of alveoli required for the first breath after birth [20]–[22]. Lung alveoli are lined by type I and II pneumocytes that are required for gas exchange and surfactant production to reduce surface tension, respectively. Failure of the lung to expand after the first breath is one of the most common causes of neonatal morbidity and mortality from diseases such as infant respiratory distress syndrome [22], [23]. Among the surfactant proteins expressed in type II cells, including surfactant protein A (SP-A), SP-B, SP-C, and SP-D [24], [25], SP-B is required for postnatal lung function and survival [26]–[28]. Complete deficiency of *SP-B* in mice and humans results in lethal neonatal RDS, which is characterized by a virtual absence of lung compliance and increased amounts of incompletely processed proprotein SP-C [26], [27], [29]–[31]. Loss or partial reduction of SP-B expression has been observed in patients without *SP-B* gene mutation [27], but whether such losses or reductions in *SP-B* expression are associated with epigenetic alterations remains largely unknown.

To investigate the function and mechanisms of Jmjd3 *in vivo*, we generated *Jmjd3* global and tissue-specific knockout mice. We found that *Jmjd3* ablation induces perinatal lethality associated with respiratory failure caused by defective lung development. Tissue-specific deletion of *Jmjd3* and tamoxifen (TM)-induced temporal deletion revealed Jmjd3 is dispensable in the later embryonic development (after E9.5) stages. Gene expression profiling, tissue staining, and chromatin immunoprecipitation-sequencing (ChIP-Seq) analyses showed that *Jmjd3* deficiency markedly reduces a set of genes critical for lung development, in particular *SP-B*. Jmjd3-mediated changes in target gene expression are associated with alterations in H3K27 and H3K4 methylation levels in the proximal promoter region. Jmjd3 regulates *SP-B* expression in a locus-specific manner through interactions with the transcription factor Nkx2.1 and the epigenetic protein Brg1. Together, our findings show that Jmjd3 plays a stage-dependent and locus-specific role during embryonic lung development. This function of Jmjd3 is associated with the epigenetic regulation of lung surfactant protein gene expression.

## Results

### *Jmjd3* deletion causes multiple embryonic defects

**Figure 1:**
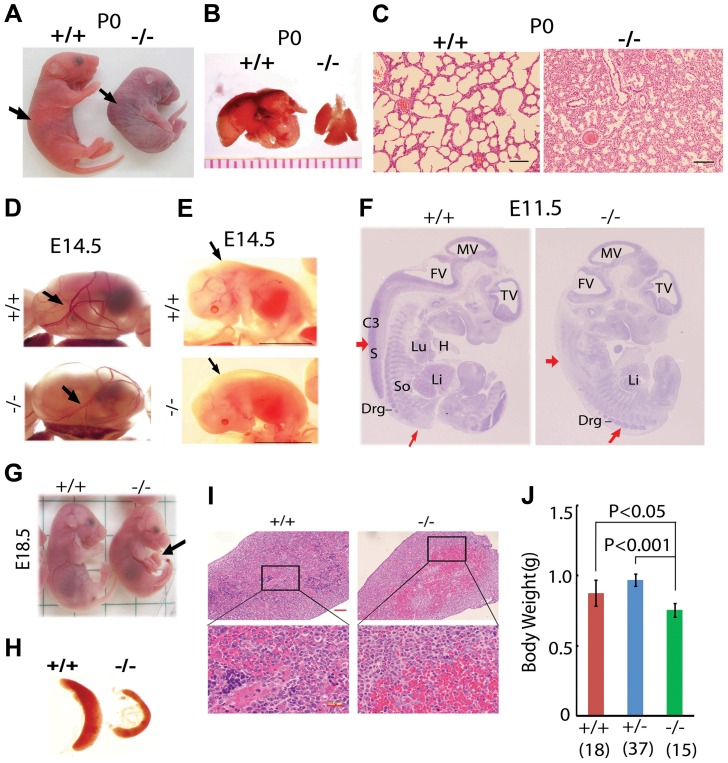
Postnatal lethality and multiple organ defects of *Jmjd3* ^−/−^ embryos. (**A**) Postnatal lethality of *Jmjd3* ^−/−^ mice. The skin color of *Jmjd3* ^−/−^ mice became cyanotic minutes after birth. Arrowhead indicates defective lordosis in *Jmjd3* ^−/−^ newborns. (**B**) The lungs of *Jmjd3* ^−/−^ mice were smaller and not expanded at P0 compared with *Jmjd^+/+^* mice. (**C**) H&E staining of P0 lung sections from *Jmjd3* ^−/−^ mice indicates arrested tissue maturation. (**D**) Less developed yolk sac vascular plexus of *Jmjd3* ^−/−^ embryos at E14.5. (**E**) Subcutaneous edema on the dorsal region of *Jmjd3* ^−/−^ embryos at E14.5. (**F**) H&E staining of sagittal sections of *Jmjd3* ^−/−^ embryos at E11.5 indicating defective spinal cord and somite development. S, spinal cord; So, somite; Drg, dorsal root ganglion; H, heart; Lu, lung; Li, liver; C3, centrum of C3; TV, third ventricle; FV, fourth ventricle; MV, mesencephalic vesicle. (**G**) Failure of umbilical cord resorption (arrowhead) in E18.5 *Jmjd3* ^−/−^ embryos. (**H**) The spleens of *Jmjd3*-deficient embryos at E17.5 were smaller than those of WT embryos. (**I**) H&E staining showing multiple hyperemic areas in the spleen of *Jmjd3* ^−/−^ embryos. (**J**) Body weight of *Jmjd3* ^−/−^ embryos was significantly lower than that of WT and *Jmjd3* ^+/−^ controls. Data are presented as the mean ± SD. Differences in body weight were analyzed using the Student's *t*-test.

To study the *in vivo* functions of Jmjd3 during development, *Jmjd3* KO mice were generated by homologous recombination technique [17]. Heterozygous *Jmjd3^+/−^* mice were fertile and viable. However, homozygous *Jmjd3*-deficient mice were not recovered at weaning time (P21) from the heterozygous *Jmjd3* ^+/−^ mating pairs (Table S1), suggesting that *Jmjd3* deficiency induces postnatal lethality. To confirm this, the embryos were genotyped and found to be consistent with a Mendelian ratio distribution at the late gestation stages (E11.5, E14.5, E17.5, E19.5) up to postnatal day 0 (P0). However, homozygous *Jmjd3*-deficient mice died shortly after birth (P0) with kyphosis and severe lordosis (Figure 1A). *Jmjd3*-deficient newborns and their wild-type (WT) littermates had pink skin color at birth; however, *Jmjd3*-deficient newborns became cyanotic within minutes due to respiratory failure (Figure 1A). Consistent with this observation, we found that the lungs of *Jmjd3*-deficient mice were not inflated with air and much smaller in size compared with the lungs of WT mice (Figure 1B). Hematoxylin & eosin (H&E) staining revealed that *Jmjd3*-deficient lungs were arrested in the late canalicular stage with limited sac spaces, undilated acinar bibules, and buds apparent in the peripheral regions. In contrast, WT lungs contained well-developed pre-alveoli and thinned out mesenchyme (Figures 1C and S1A). The alveolar sacs and associated capillary beds were less developed in *Jmjd3*-deficient lungs compared with WT lungs. At E17.5, *Jmjd3*-deficienct lungs were smaller with less developed lobes compared with WT lungs (Figure S1B). H&E staining revealed the severe development defects (Figure S1C). Together, these observations indicate that Jmjd3 is essential for lung development.

In addition to the lung, *Jmjd3* deficiency caused other embryonic defects. The yolk sac vascular plexus was less developed in *Jmjd3*-deficient embryos than in WT embryos at E14.5 (Figure 1D). *Jmjd3* ^−/−^ embryos developed subcutaneous edema in the upper back at E14.5, reflecting defective muscle and skeletal development (Figure 1E). Consistent with these observations, H&E staining of sagittal sections of *Jmjd3*-deficient E11.5 embryos revealed multiple developmental defects, including delayed ganglia, somite, and spinal cord development (Figure 1F). We also observed that the umbilical hernia at E18.5 was not absorbed in *Jmjd3*-deficient mice, which is hazardous for newborn mice survival (Figure 1G). Because Jmjd3 has been associated with immune cell-mediated inflammation [32], we examined the spleens of *Jmjd3*-deficient and WT embryos. The spleens of *Jmjd3*-deficient embryos were smaller and had multiple hyperemic areas compared with WT embryos (Figure 1H). H&E staining also revealed the accumulation of blood cells in *Jmjd3*-deficient spleens (Figure 1I). *Jmjd3*-deficient mice were also markedly smaller than their WT littermates at birth (Figure 1A, 1J). These results indicate that Jmjd3 plays an important role in embryonic development.

### Tissue-specific deletion of Jmjd3 in lung development

**Figure 2:**
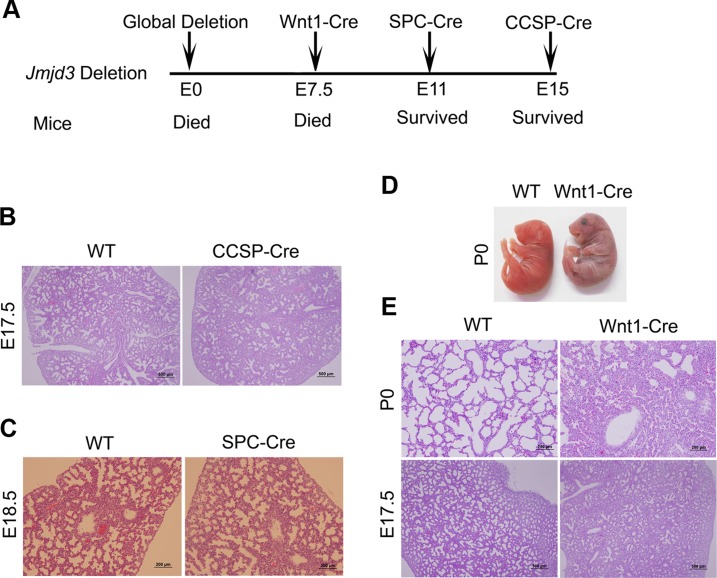
Effect of tissue-specific Jmjd3 deletion on mouse survival and lung development. (**A**) Schematic drawing showing the effects of tissue-specific Cre expression on mouse survival. (**B**) H&E staining of lung tissues from *Jmjd3^f/f^:CCSP-Cre* and WT embryos at E17.5. (**C**) H&E staining of lung tissues from *Jmjd3^f/f^:SPC-Cre* and WT embryos at E18.5. (**D**) Postnatal lethality (within 8 h) was observed in *Jmjd3^f/f^:Wnt1-Cre* embryos. (**E**) H&E staining of lung tissues from *Jmjd3^f/f^:Wnt1-Cre* and WT embryos at P0 and E17.5.

Because *Jmjd3*-deficient newborn mice died shortly after birth of respiratory failure, we sought to determine whether tissue-specific ablation of *Jmjd3* recapitulates the global deletion phenotype. To do this, we first crossed *Jmjd3^f/f^* mice with *CCSP-Cre* mice in which *Cre* is expressed under the control of the *CCSP* promoter in Clara cells at early E15 [33]. Lung structure was similar between *Jmjd3^f/f^*:*CCSP-Cre* and WT mice (Figure 2A, 2B), suggesting that *Jmjd3* may not be important in Clara cells during lung development after E15. Next, we crossed *Jmjd3^f/f^* mice with *SPC-Cre* mice in which the *SPC* promoter drives the expression of Cre recombinase gene in distal progenitor cells at E11.5 [34]. We found that *Jmjd3^f/f^*:*SPC-Cre* mice were viable from birth to adulthood without any dramatic defects in the lung architecture (Figure 2A, 2C). To rule out the possibility that the H3K27me2/3 demethylase UTX compensates for the loss of Jmjd3 function in *Jmjd3* deficient mice, we generated both *Jmjd3* and *UTX* specific deletion mice by crossing *Jmjd3^floxed^* and *Utx^floxed^* with SPC-Cre mice. *Jmjd3^f/f^:Utx^f/f^:SPC-Cre* mice exhibit a similar phenotype to that of *Jmjd3^f/f^:SPC-Cre* pups, indicating that UTX does not compensate for the loss of Jmjd3 (Figure S2).

Because *SPC* and *CCSP*-driven expression of Cre are detectable after lung development initiation [35], [36], we reasoned that Jmjd3 is not critical in epithelial cells once lung development is initiated. To test this possibility, *Jmjd3^f/f^* mice were crossed with *Wnt1-Cre* mice in which Cre expression is driven by the *Wnt1* gene promoter [37], [38]. The Wnt1 protein is predominantly expressed in the inner cell mass of the blastocyst during the early stages of embryonic development (E7.5) [39]. Wnt1-driven Cre is mainly expressed in neural crest cells [38] and intrinsic innervation of the lung [37]. Intrinsic nerve ganglia, which are derived from neural crest cells, are required for normal lung development and function [36]. Although the Wnt1-Cre model is often used to study neural crest formation, it is also used to study gene function during organogenesis in mice [39]. We found that all *Jmjd3^f/f^*:*Wnt1-Cre* newborn pups died within 8 h of respiratory failure (Figure 2D). H&E staining showed that lung structure was severely affected with fewer sac spaces and undilated acinar bibules at different embryonic stages (Figure 2E), suggests the potential involvement of Jmjd3 in the early embryonic stage of lung development.

### Jmjd3 is dispensable for lung development at E9.5 or later embryonic stages

**Figure 3:**
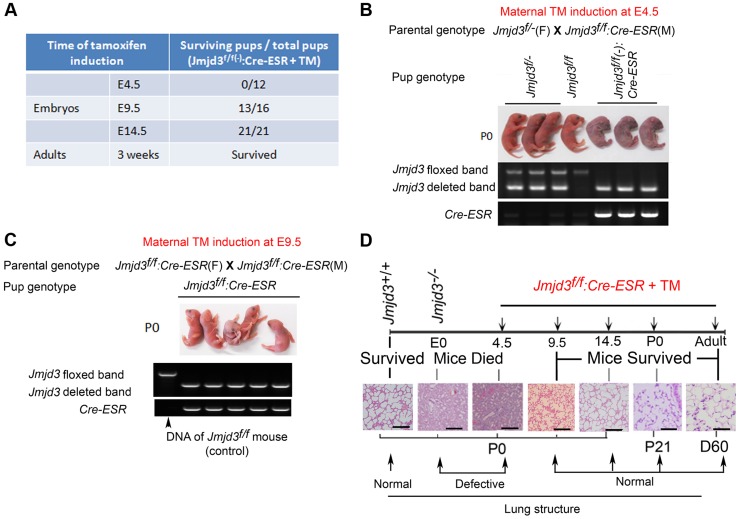
Stage-specific deletion reveals that Jmjd3 is dispensable for lung development in later E9.5 embryonic stage. (**A**) *Jmjd3^f/f^* mice were crossed with *CAG-Cre/ESR* mice in which Cre expression is globally induced with TM. The survival of *Jmjd3^f/f^:Cre/ESR* pups from maternal mice treated with TM at E4.5, E9.5, and E14.5 was determined. (**B**) PCR analysis of *Jmjd3* deletion efficiency in *Jmjd3^f/f^:Cre/ESR* pups from maternal mice treated with TM at E4.5. (**C**) PCR analysis of *Jmjd3* deletion efficiency in *Jmjd3^f/f^:Cre/ESR* pups from maternal mice treated with TM at E9.5. (**D**) H&E staining showing the stage-dependent effects of *Jmjd3* deletion on lung architecture and its correlation with embryo viability. Bar = 500 µm.

Because Jmjd3 is required for the early embryonic lung development, we further studied the importance of *Jmjd3* at different embryonic stages. To do this, we crossed *Jmjd3^f/f^* mice with *CAG-Cre/ESR* mice in which Cre expression is globally induced with TM. *Jmjd3^f/f^*:*CAG-Cre/ESR* offspring from TM-treated maternal mice at E4.5 stage died shortly after birth and exhibited a phenotype similar to that of *Jmjd3*-deficienct embryos, whereas *Jmjd3^f/f^* and WT offspring of the same litter survived (Figure 3A, 3B). In contrast, the survival rate was increased in *Jmjd3^f/f^*:*CAG-Cre/ESR* offspring from TM-treated maternal mice at E9.5 or later stages (Figure 3A, 3C). Polymerase chain reaction (PCR) analysis showed *Jmjd3* deletion in the lethal and surviving *Jmjd3^f/f^*:*CAG-Cre/ESR* pups (Figure 3B, 3C). H&E staining showed that the lung structure of *Jmjd3^f/f^;CAG-Cre/ESR* offspring from TM-treated maternal mice at E4.5 was comparable to that of *Jmjd3^−/−^* newborns, whereas lung structure was similar between *Jmjd3^f/f^:CAG-Cre/ESR* surviving offspring from TM-treated maternal mice at E9.5 or later and WT mice (Figure 3D). The lungs of *Jmjd3^f/f^:CAG-Cre/ESR* adult mice treated with TM did not show any structural abnormalities (Figure 3D). Taken together, these findings indicate that *Jmjd3* is dispensable in the later E9.5 embryonic stage of lung development.

### Identification of the surfactant protein family as Jmjd3 targets in lung tissue

**Figure 4:**
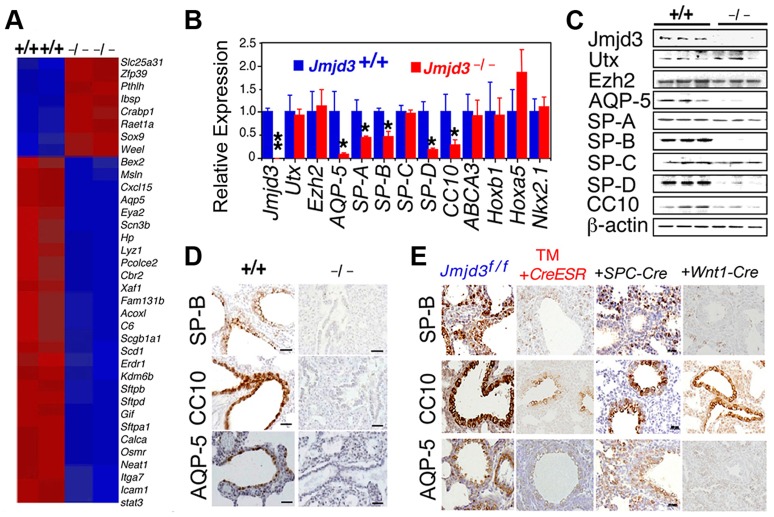
Jmjd3 regulates the expression of genetic markers of lung development. (**A**) Microarray analysis showing representative genes that were differentially expressed between WT and *Jmjd3* ^−/−^ lung tissues at E17.5. Red, upregulated genes; blue, downregulated genes. (**B**) qPCR was done to validate the expression of selected genes in WT and *Jmjd3* ^−/−^ lung tissues. Gene expression levels were normalized to β-actin. Gene expression levels in WT lung tissue were defined as 1. Data are presented as the mean ± SD. *P<0.05 and **P<0.001 (Student's *t* test). (**C**) Immunoblot analysis of the protein expression of selected genes in the lung tissues of *Jmjd3* ^+/+^ and *Jmjd3* ^−/−^ embryos at E19.5. (**D**) Immunohistocytochemical staining of SP-B, CC10, and AQP-5 protein expression in the lung tissues of *Jmjd3* ^+/+^ and *Jmjd3* ^−/−^ embryos at E19.5. Data shown are representative of three independent experiments. Bar = 50 µm. (**E**) Immunohistocytochemical staining of SP-B, CC10, and AQP-5 expression in the lungs of *Jmjd3^f/f^*, *Jmjd3^f/f^:CreESR*/TM, *Jmjd3^f/f^:SPC-Cre*, and *Jmjd3^f/f^:Wnt1-Cre* mice. Data shown are representative of three independent experiments. Bar = 50 µm.

Having established a role for Jmjd3 in lung development, we next investigated the underlying molecular mechanisms by identifying the target genes of Jmjd3 in lung tissue. Affymetrix microarray analysis was performed on RNA samples isolated from WT and *Jmjd3* ^−/−^ E17.5 lungs. Of the 45,000 probes analyzed, 244 genes were downregulated and 190 genes were upregulated in *Jmjd3*-deficient lung tissues compared with WT lung tissues (Table S2). Representative genes with altered expression (>2 fold) are shown in Figure 4A. Importantly, *Jmjd3* deficiency downregulated the expression of genes known to play important roles in lung development and function, including *aquaporin-5* (*AQP-5*), *SP-A*, *SP-B*, *SP-D*, and *Clara cell 10 kDa secretory protein* (*CC10*). Consistent with this observation, qPCR and immunoblot analyses showed that AQP-5, SP-B, SP-D, and CC10 mRNA and protein expression were markedly reduced in *Jmjd3*-deficient lung tissues compared with WT lung tissues (Figure 4B, 4C). These results were further supported by histological analysis of AQP-5, SP-B, and CC10 protein expression in *Jmjd3*-deficient lung tissues (Figure 4D). *SP-A* RNA, but not protein, expression was significantly reduced in *Jmjd3*-deficient lung tissues compared with WT controls, whereas *SP-C* expression was not appreciably different (Figure 4B, 4C). Although UTX and Ezh2 are involved in the control of H3K27 methylation, their expression levels were not affected by *Jmjd3* deficiency in lung tissues (Figure 4B, 4C). As a recent report implicated respiratory rhythm generator (RRG) and pre-Bötzinger complex dysfunction in the impaired respiratory function of *Jmjd3* deficient mice [19], we analyzed the expression of RRG-related and pre-Bötzinger complex genes in *Jmjd3*-deficient brainstem and lung tissues. qPCR analysis showed that the expression of several pre-Bötzinger complex-specific genes including *Esrrg*, *Kirrel3*, and *March4* was unchanged at E17.5, but increased rather than decreased at P0 in the *Jmjd3* deficient brainstem compared with the WT brainstem (Figure S3A). Furthermore, the expression of these pre-Bötzinger complex-specific genes was not different between *Jmjd3*-deficient and WT E17.5 lung tissues (Figure S3B). Respiratory muscles, including diaphragm and intercostal muscles, play an important role in breathing-like movement. Expression analysis of genes in diaphragm development including *Fog2*, *COUP-TFII* and *Wt1* by qPCR did not reveal a significant difference between WT and *Jmjd3*-deficient mice (Figure S3C).

To determine the stage-specific requirement of Jmjd3 in the regulation of lung development genes, we used the TM inducible and Wnt1-Cre deletion models. SP-B, CC10, and AQP-5 immunostaining were negative in the lung tissues of *Jmjd3^f/f^*:*CAG-Cre/ESR* offspring from TM-treated maternal mice at E4.5 but were readily detectable in the lung tissues of *Jmjd3^f/f^* offspring from similarly treated maternal mice (Figure 4E). This finding indicates that *Jmjd3* deletion at E4.5 effectively reduces SP-B, CC10, and AQP-5 expression in lung tissues, leading to defects in lung development and function. SP-B and AQP-5 immunostaining were also dramatically decreased in lung tissues of *Jmjd3^f/f^*:*Wnt1-Cre* mice at P0 (Figure 4E). Together, these results suggest that *Jmjd3* ablation specifically reduces the expression of *AQP-5*, *SP-B*, *SP-D*, and *CC10* genes in lung tissues and underscores the importance of Jmjd3 in the temporal and spatial regulation of genes that are critically required for lung development during embryonic development.

### Regulation of target gene expression by Jmjd3 through locus-specific histone demethylation

Jmjd3 functions as a H3K27 demethylase *in vitro* [7]–[11]; therefore, we determined whether *Jmjd3* ablation affects H3K27 methylation *in vivo* in the lung. Global H3K27 di- and trimethylation (H3K27me2 and H3K27me3, respectively) were markedly increased in *Jmjd3*-deficient lungs compared with WT lungs (Figure S4A). H3K4me3 was also slightly higher in *Jmjd3*-deficient lungs than in WT lungs, whereas H3K9 methylation was similar between *Jmjd3*-deficient and WT lungs (Figure S4A). We also examined the methylation status in *Jmjd3*-deficient mouse embryonic fibroblasts (MEFs) by immunofluorescence staining and found increased H3K27me2 and H3K27me3 in *Jmjd3^−/−^* MEFs (Figure S4B). Furthermore, immunoblot analysis also revealed that both H3K27me2 and H3K27me3 were markedly increased in *Jmjd3^−/−^* MEFs compared with WT MEFs, whereas appreciable differences in the methylation of H3K4 and H3K9 were not observed (Figure S4C). To address whether the increase in H3K27 di- and trimethylation was directly related to *Jmjd3* deletion, we examined the methylation status of *Jmjd3*-deficient MEFs transiently transfected with a FLAG-tagged *Jmjd3*-expressing retrovirus. H3K27 dimethylation and trimethylation were decreased in FLAG-tagged *Jmjd3*-overexpressing *Jmjd3^−/−^* MEFs (Figure S4D), suggesting that *Jmjd3* deletion alters global H3K27 methylation levels in MEFs and lung tissues.

**Figure 5:**
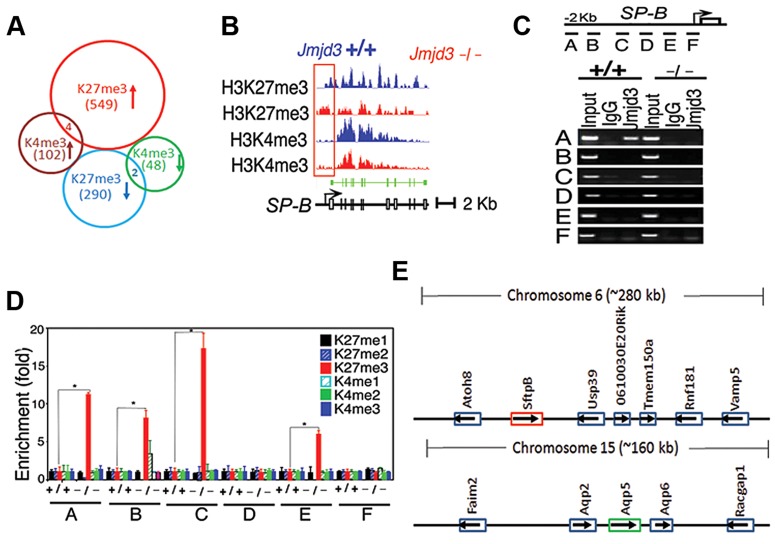
*Jmjd*3 ablation affects global histone methylation in lung tissues and methylation status of the promoter regions of target genes. (**A**) Global gene methylation analysis of *Jmjd3* ^+/+^ and *Jmjd3* ^−/−^ lung tissues at E17.5 by ChIP-Seq. ↑, methylation increased; ↓, methylation decreased. (**B**) ChIP-Seq analysis of H3K27me3 and H3K4me3 levels in the promoter and gene body regions of the *SP-B* gene in *Jmjd3* ^+/+^ and *Jmjd3* ^−/−^ lung tissues at E17.5. Data shown are representative of three independent experiments. (**C**) Jmjd3 binding to the SP-B promoter in lung tissues was determined by ChIP-PCR. Chromatin was immunoprecipitated from the lung tissues of E17.5 *Jmjd3* ^+/+^ and *Jmjd3* ^−/−^ embryos. Primer design for the ChIP-PCR assay of mouse *SP-B* promoter regions (*top panel*). The primers sets cover the following regions: A, −2347–−2142; B, −2065–−1835; C, −1451–−1334; D, −1001–−878; E, −516–−383; F, −218–+14. ChIP-PCR assay showing Jmjd3 binding around 2 kb upstream of the TSS of the *SP-B* promoter region (*bottom panel*). (**D**) ChIP-qPCR analysis of histone methylation levels in the *SP-B* promoter region in the lung tissues of E17.5 *Jmjd3* ^+/+^ and *Jmjd3* ^−/−^ embryos. (**E**) Locus-specific demethylation analysis of Jmjd3 by ChIP-Seq. ChIP-Seq was done to determine the H3K27 and H3K4 methylation level of genes located in the region (∼280 kb) containing *SP-B* on chromosome 6 and the region (∼160 kb) containing *AQP-5* on chromosome 15. Arrows indicate the gene expression direction.

To further define changes in the H3K4 and H3K27 methylation status of Jmjd3 target genes in lung tissue, we performed ChIP-Seq analysis on WT and *Jmjd3*-deficienct lung tissues. Among the 35,000 RefSeq genes, 553 genes had increased H3K27me3 around 5 kb of the transcription start site (TSS) and 292 genes had decreased H3K27me3 in *Jmjd3*-deficient lungs compared with WT controls (Figure 5A; Table S3). The number of genes with increased H3K4me3 was greater than the number of genes with decreased H3K4me3 in *Jmjd3*-deficient lungs (106 vs. 50; Tables S3, S4), which was consistent with the immunoblot analysis of H3K4 methylation (Figure 5A).

ChIP-Seq analysis of individual genes revealed that H3K27me3 amounts in the upstream promoter regions of the TSS of *SP-B* were markedly increased in *Jmjd3*-deficient lung tissues compared with WT controls, whereas H3K4me3 in the gene body regions of *SP-B* was slightly reduced in *Jmjd3*-deficient lung tissues (Figure 5B). The amounts of H3K4me3 around the TSS and gene body regions of *AQP-5*, *SP-D*, and *CC10* were also markedly decreased in *Jmjd3*-deficient lung tissues compared with WT controls (Figure S5A). In contrast, we did not observe any appreciable differences in H3K4 and H3K27 methylation in the promoter and body regions of *SP-C, ABCA3, Nkx2.1, Hoxb1, Hoxa5, Ezh2*, and *UTX*, whereas H3K4me3, but not H3K27me3, was slightly decreased in the *SP-A* gene (Figure S5A). ChIP-Seq analysis also revealed that H3K27me3 and H3K4me3 methylation levels in the promoter regions or gene bodies of the RRG-related genes *Phox2b*, *Tshz3*, *Task2*, *Maoa*, *Phox2a*, *Reln*, and *March4* were not different between *Jmjd3*-deficient and WT E17.5 lung tissues (Figure S5B). To further validate the ChIP-Seq results, we performed ChIP-PCR analysis on WT and *Jmjd3*-deficient lung tissues at E17.5. A strong band was detected around 2 kb upstream of the TSS of *SP-B* (Figure 5C), indicating that Jmjd3 is recruited to the regulatory region of the *SP-B* gene. In addition, Jmjd3 was also bound to the upstream regions of the TSS of *AQP-5*, *SP-A*, *SP-D*, and *CC10*, but not to those of *SP-C* (Figure S5C).

Next, we assessed the effect of Jmjd3 on the histone methylation status of the *SP-B* promoter region using ChIP-qPCR. H3K27me3 was markedly increased in the A, B, C, and E regions of the *SP-B* gene promoter in *Jmjd3*-deficient lungs compared with WT controls (Figure 5D). Increased levels of H3K27me3 were also observed in the promoter regions of *CC10* and *SP-D*, but not in those of *AQP-5*, in *Jmjd3*-deficient lungs (Figure S5D). In contrast, the methylation levels of H3K4me3 in the promoter regions (i.e., B and C regions near the TSS) of *AQP-5*, *CC10*, and *SP-D* were markedly lower in *Jmjd3*-deficient lungs than in WT controls (Figure S5D). These results indicate that *Jmjd3* ablation in lung tissues affects H3K27me3 and H3K4me3 levels in the promoter and gene body regions of target genes.

To determine whether Jmjd3-mediated changes in histone methylation status were chromosome or locus-specific, we randomly analyzed the H3K27 and H3K4 methylation level of genes located in the region (∼280 kb) containing *SP-B* on chromosome 6 and the region (∼160 kb) containing *AQP-5* on chromosome 15. Our ChIP-Seq data showed that H3K27 and H3K4 methylation of both *SP-B* and *AQP-5* were altered, whereas H3K27 and H3K4 methylation in neighbouring loci were not significantly changed. This data suggests that Jmjd3 targets specific loci for histone demethylation and does not affect histone methylation in adjacent chromosome regions (Figure 5E).

### Jmjd3 regulates target gene expression by interacting with transcription and epigenetic factors

**Figure 6:**
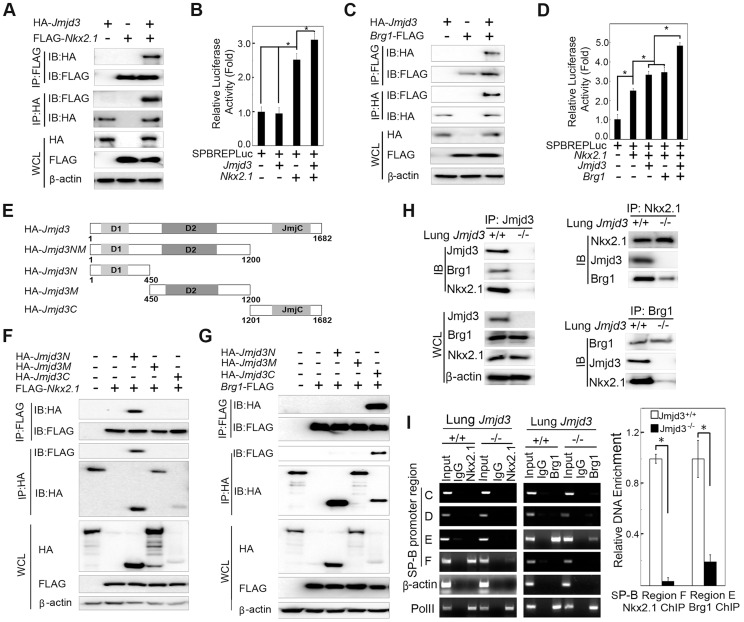
Jmjd3 coregulates *SP-B* expression with Nkx2.1 and Brg1. (**A**) The interaction between Jmjd3 and Nkx2.1 was evaluated by coimmunoprecipitation (CoIP) analysis of 293T cells expressing HA-tagged *Jmjd3* and FLAG-tagged *Nkx2.1*. (**B**) A cell-based luciferase assay was used to evaluate *SP-B* promoter activity in 293T cells cotransfected with *Nkx2.1*, *Jmjd3*, and mouse *SP-B* promoter-linked episomal luciferase vector (containing Nkx2.1 binding sites). (**C**) The interaction between Jmjd3 and Brg1 was determined by CoIP analysis of 293T cells expressing HA-tagged *Jmjd3* and FLAG-tagged *Brg1*. (**D**) Cell-based luciferase assay was used to evaluate *SP-B* promoter activity in 293T cells cotransfected with *Nkx2.1*, *Jmjd3*, *Brg1*, and mouse *SP-B* promoter-linked episomal luciferase vector. *P<0.05 (Student's *t* test). (**E**) Generation of *Jmjd3* deletion constructs. Numbers represent the corresponding amino acid residue position in the *Jmjd3* coding region. (**F**) CoIP analysis of the interaction between Nkx2.1 and HA-tagged truncated Jmjd3 proteins. (**G**) CoIP analysis of the interaction between Brg1 and truncated Jmjd3 proteins. (**H**) CoIP analysis of the interaction among endogenous Nkx2.1, Brg1, and Jmjd3 in WT and *Jmjd3*-deficient lung tissues. (**I**) ChIP-PCR and ChIP-qPCR analysis of Nkx2.1 and Brg1 binding to *SP-B* promoter regions in WT and *Jmjd3*-deficient lung tissues. *P<0.05 (Student's *t* test).

Jmjd3 is a general H3K27 demethylase without specific DNA binding properties and the mechanisms by which it regulates specific gene expression remain unknown. Therefore, we reasoned that Jmjd3 might be recruited to specific gene promoters through interactions with key transcription factors and/or epigenetic proteins. To test this possibility, we first determined whether Jmjd3 regulates target gene expression by interacting with Nkx2.1. Nkx2.1 (also known as thyroid transcription factor 1) is a key transcriptional activator of *SP-B* gene expression [40]. Coimmunoprecipitation and immunoblot analyses revealed that Jmjd3 interacted with Nkx2.1 in 293T cells expressing HA-tagged *Jmjd3* and FLAG-tagged *Nkx2.1* (Figure 6A). To determine whether Jmjd3 affects the transcriptional activity of Nkx2.1, we performed a luciferase assay on 293T cells cotransfected with *Nkx2.1*, *Jmjd3*, and mouse *SP-B* promoter-linked episomal luciferase vector (containing Nkx2.1 binding sites) [40], [41]. Jmjd3 alone did not affect luciferase activity; however, it significantly increased the ability of Nkx2.1 to enhance SP-B-mediated luciferase activity (Figure 6B). In contrast, an interaction between exogenous UTX and Nkx2.1 was not observed in 293T cells (Figure S6). Thus, the Nkx2.1 interaction appears to be specific to histone demethylase Jmjd3. These findings suggest that Jmjd3 regulates *SP-B* expression by interacting with Nkx2.1 in the *SP-B* promoter region.

Brg1, an ATPase subunit of the Swi/Snf chromatin remodeling complex, has been shown to cooperate with Nkx2-1 to regulate *SP-B* expression [40]. Jmjd3 interacted with Brg1 in 293T cells expressing HA-tagged *Jmjd3* and FLAG-tagged *Brg1* (Figure 6C), consistent with the results of a previous study [42]. Furthermore, Jmjd3 significantly enhanced Nkx2.1 and Brg1-mediated *SP-B* promoter activity (Figure 6D). To determine the region of Jmjd3 that interacts with Nkx2.1 and Brg1, we generated four HA-tagged Jmjd3 truncated constructs: Jmjd3N (1–450 aa), Jmjd3M (450–1200 aa), Jmjd3C (1201–1682 aa), and Jmjd3NM (1–1200 aa) (Figure 6E). The N-terminus of Jmjd3 (Jmjd3N) strongly interacted with Nkx2.1, whereas the C-terminus of Jmjd3 (Jmjd3C) specifically interacted with Brg1 (Figure 6F, 6G). Importantly, the endogenous interaction of Jmjd3 with Nkx2.1 and Brg1 was readily detected in WT, but not in *Jmjd3*-deficient, lung tissues at E17.5 (Figure 6H), suggesting that Jmjd3 may be required for Nkx2.1/Brg1 complex formation on the *SP-B* promoter region. However, the interaction between Nkx2.1 and Brg1 was still detected in *Jmjd3*-deficient lung tissues, although to a lesser extent compared with WT tissues (Figure 6H). Therefore, Jmjd3 may also influence the interaction between Nkx2.1 and Brg1. The role of Jmjd3 in the Nkx2.1/Brg1 interaction was confirmed by ChIP-qPCR, which showed a marked reduction in Nkx2.1 and Brg1 binding to *SP-B* promoter regions in *Jmjd3*-deficient lung tissues (Figure 6I). Taken together, our results suggest that Jmjd3 activates *SP-B* expression by specifically interacting with Nkx2.1 and Brg1 to form Nkx2.1-Brg1-Jmjd3 complexes.

### Regulation of *SP-B* expression by Jmjd3 is dependent on its H3K27 demethylase activity

**Figure 7:**
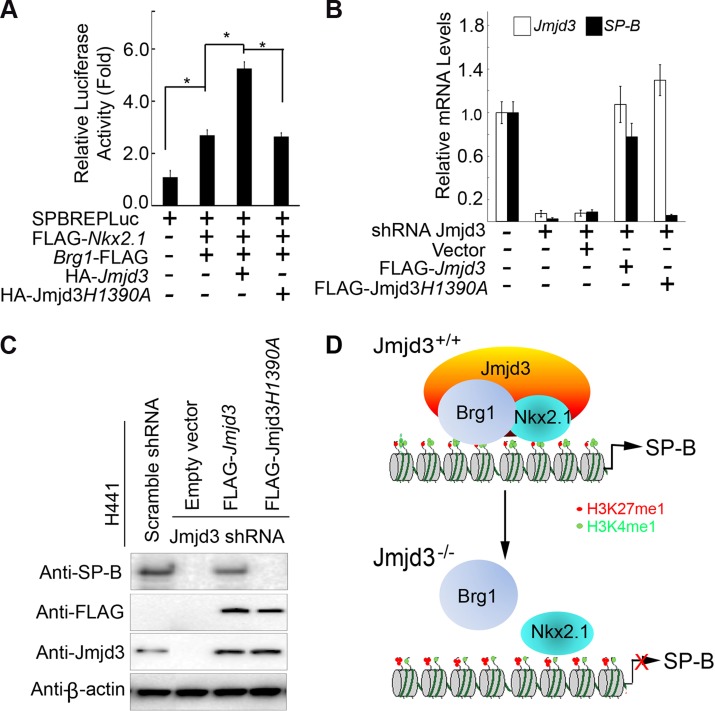
The demethylase activity of Jmjd3 is required for the regulation of *SP-B* expression. (**A**) WT, but not mutant, Jmjd3 enhanced NKx2.1 and Brg1-mediated *SP-B* promoter activity. Data are presented as the mean ± SD from three independent experiments. *P<0.05 (Student's *t* test). (**B**) qPCR analysis of *SP-B* expression in Jmjd3-specific shRNA-expressing H441 cells transfected with WT or mutant *Jmjd3*. Data are presented as the mean ± SD from three independent experiments. (**C**) Immunoblot analysis of SP-B protein in Jmjd3-specific shRNA-expressing H441 cells transfected with WT or mutant *Jmjd3*. (**D**) A proposed model explaining how Jmjd3 specifically upregulates SP-B expression by interacting with Nkx2.1 and Brg1 in the SP-B promoter.

We next asked whether the demethylase activity of Jmjd3 is required for regulating *SP-B* expression. To address this, a luciferase assay was performed on 293T cells cotransfected with *SP-B* promoter-linked episomal luciferase vector, *Nkx2.1*, and *Brg1* in the presence of WT or mutant *Jmjd3* (a loss of function mutation in H1390A in the catalytic domain of the demethylase). WT *Jmjd3* significantly enhanced Nkx2.1 and Brg1-mediated *SP-B* promoter activity, whereas mutant *Jmjd3* failed to do so (Figure 7A). To further test the ability of mutant *Jmjd3* to rescue SP-B expression, WT and mutant *Jmjd3* constructs were ectopically expressed in human H441 cells stably transfected with *Jmjd3*-specific shRNA. WT, but not mutant, *Jmjd3* rescued endogenous SP-B mRNA and protein expression (Figure 7B, 7C). We next determined whether the catalytic domain (located at the C-terminus) of Jmjd3 was sufficient to regulate *SP-B* expression. Full-length Jmjd3 markedly enhanced the luciferase activity of the *SP-B* promoter compared with truncated Jmjd3 containing only the catalytic domain (Figure S7). Taken together, these results suggest that full-length Jmjd3 is required for regulating *SP-B* expression in a demethylase activity-dependent manner.

## Discussion

In this study, we show that Jmjd3 is important for organogenesis during embryonic development, as evidenced by the multiple organs defects induced by global *Jmjd3* deficiency. The importance of Jmjd3 in embryonic lung development appears to be associated with its epigenetic regulation of target genes required for lung development and function. However, our results obtained from lung epithelial cell-specific deletion of *Jmjd3* crossed with SPC-Cre and CCSP-Cre mice suggest *Jmjd3* is dispensable for lung development at E9.5 or later embryonic stages. This notion is further supported by results obtained with Jmjd3 deletion in TM-inducible Cre mouse models. To investigate whether Jmjd3 plays a role in the lung mesenchyme or the communication between the mesenchyme and epithelium cells, further experiments using other Cre mice such as Dermo-Cre for *Jmjd3* deletion are needed and currently under way. Our study identifies key target genes including *SP-B*, *CC10* and *AQP-5*, involved in lung development and function. Changes in jmjd3 target gene expression are associated with locus-specific methylation alterations of H3K27 and H3K4, providing new insights into the role and molecular mechanism of action of Jmjd3 in embryonic lung development.

Our results show that the global deletion of *Jmjd3* results in perinatal lethality in mice shortly after birth. Similarly, several studies have also shown that *Jmjd3* deficiency is associated with a lethal phenotype in mice; however, the severity of the lethal phenotype varies according to the deletion strategy used to generate *Jmjd3* KO mice [15], [18], [19]. In the present study, deletion of the demethylase catalytic domain of *Jmjd3* induces perinatal lethality 30 min after birth. Satoh et al. [15] reported similar findings with conventional deletion of the same Jmjd3 catalytic domain. In both our own and the Satoh et al. [15] studies, the perinatal lethality of the *Jmjd3* global deletion phenotype is associated with respiratory failure caused by defects in lung development. By contrast, *Jmjd3* deletion mice generated using a gene trap strategy to insert a neo-cassette between exons 1 and 2 exhibit postnatal lethality, some pups survive up to one day [19]. The surviving pups may be attributed to the leaky expression of *Jmjd3* associated with the gene-trap strategy. Despite similar postnatal lethality caused by respiratory failure, lung development and structure are normal in the Jmjd3 TRAP insertion mice [19], suggesting that two inactivation strategies (targeted deletion of functional domain and gene-trap) show different phenotypes in lung development and function. Furthermore, Ohtani et al. [18] showed that mice with complete abrogation of Jmjd3 expression through a deletion (exons 4 to 5)-induced frameshift exhibit a more severe phenotype characterized by embryonic lethality before E6.5. Taken together, these studies show that mice generated by different *Jmjd3* deletion strategies develop different phenotypes.

It is likely that *Jmjd3* deletion causes multiple defects in different organs or tissues by affecting different Jmjd3 target genes through different mechanisms. In our study, *Jmjd3*-deficient mice die shortly after birth of respiratory failure associated with defective lung development. Consistent with our findings, Satoh et al. [15] showed that conventional deletion of the Jmjd3 catalytic domain results in premature lung development in mice; however, target genes and mechanistic study of Jmjd3 in lung development was not provided in their study. In a recent study, Burgold et al. [19] also found that the perinatal lethality of *Jmjd3* deficiency is caused by respiratory failure. However, lung development was normal in *Jmjd3*-deficient mice, and postnatal lethality was associated with Jmjd3-mediated disruption of the embryonic respiratory neuronal network and RRG. To determine whether *Jmjd3* deficiency affects RRG-related or pre-Bötzinger complex gene expression in lung tissues at E17.5, we fail to provide evidence that RRG-related or pre-Bötzinger complex gene expression is altered in our *Jmjd3*-deficient mice. Consistent with these findings, *Jmjd3* deficiency does not affect H3K27me3 or H3K4me3 methylation levels in either the promoter regions or gene bodies of RRG-related genes. Furthermore, we show no appreciable difference in the expression of *Fog2*, *COUP-TFII* and *Wt1* genes in respiratory muscles at P0 between WT and *Jmjd3*-deficient mice. Different phenotypes observed in different *Jmjd3* inaction mice might be due to differences in leaky expression, targeted deletion of catalytic domain or whole coding region. It has been demonstrated that Jmjd3 affects different target gene expression or proteins through demethylase activity-dependent and independent mechanisms [15], [17], [43]. Taken together, these studies suggest that more than one mechanism is responsible for the observed different phenotypes.

Our findings presented here indicate that *Jmjd3* deficiency downregulates a set of genes including *SP-B* critical to lung maturation and function at E17.5. SP-B, a surfactant expressed in type II cells, is required for postnatal lung function and survival [26]–[28]. Complete *SP-B* deficiency in mice and humans results in lethal, neonatal RDS [23], which is characterized by a virtual absence of lung compliance and increased amounts of incompletely processed proprotein SP-C [26], [27], [29]–[31]. Similar to the *Jmjd3* deletion phenotype, *SP-B*-deficient mice die of respiratory failure immediately after birth [30]. Together, these findings indicate that the impaired respiratory function in the *Jmjd3* deletion phenotype is associated with Jmjd3-mediated regulation of lung-specific gene expression. To provide definitive evidence that the phenotype of *Jmjd3*-deficient mice is caused by downregulation of *SP-B* gene, experiments using *Jmjd3*-deficient mice crossed with *SP-B*-inducible mice in a *SP-B* deficient background are under way to determine whether inducible SP-B expression could rescue the phenotype of *Jmjd3*-deficient mice.

Since Jmjd3 has been shown to regulate gene expression in a demethylase activity-independent [43] and -dependent manner [15], we determine whether the demethylase activity of Jmjd3 is required for the regulation of target gene expression. *Jmjd3* deletion alters the H3K27 and H3K4 methylation levels in the promoter and gene body regions of a select set of target genes associated with lung function and development. A strong correlation between H3K4 and H3K27 methylation level and *AQP*-5, *SP-B*, *SP-D*, and *CC10* expression is also observed, whereas a weak correlation is observed in many other genes. Our findings are consistent with a previous study showing that changes in H3K27me3 level and gene expression are associated in a limited number of genes in *Jmjd3*-deficient macrophages [15]. *SP-B* deficiency caused by genetic mutations has been implicated as the principal cause of infant RDS; however, loss or partial reduction of SP-B expression has also been observed in patients without *SP-B* gene mutations [27]. These observations suggest the potential involvement of epigenetic mechanisms in surfactant expression. Using ChIP-qPCR, we found that *Jmjd3* deficiency markedly increases H3K27me3 at the *SP-B* promoter in lung tissues. Thus, *Jmjd3* deletion may lead to the loss of SP-B epigenetic regulation. The chromatin modifier Brg1 has been shown to cooperate with Nkx2-1 to regulate *SP-B* and *SP-A* expression [40]. Consistent with these reports, we found that Jmjd3 specifically upregulates *SP-B* expression by interacting with Nkx2-1 and Brg1.

Our ChIP-Seq analysis also indicates that Jmjd3 demethylation is locus-dependent. There are several possible reasons for this. First, the H3K27 demethylase UTX may functionally overlap with Jmjd3 and control methylation in the same or different set of genes. A recent study showed that UTX deletion results in embryonic lethality associated with cardiac defects [44]. In our study, we found that UTX does not compensate for the loss of Jmjd3 as evidenced by the similar phenotype between *Jmjd3^f/f^:Utx^f/f^:SPC-Cre* and *Jmjd3^f/f^:SPC-Cre* mice. Furthermore, Jmjd3, but not UTX, specifically interacts with Nkx2.1 to regulate *SP-B* expression, indicating that these two demethylases regulate distinct subsets of genes. Second, recent studies have indicated that the methylation status of enhancer regions is critically important in the control of gene expression [45], [46]. Thus, Jmjd3 may also control gene expression by affecting H3K27me3 level in gene enhancer regions rather than promoter regions. Third, similar to UTX, Jmjd3 may also form a complex with other histone modification proteins, such as H3K4 methyltransferases [32], [47]. Thus, *Jmjd3* deletion may potentially affect both H3K27 and H3K4 methylation in the promoter and gene-body regions of target genes, which is supported by our results. Finally, Jmjd3 is recruited to the *SP-B* promoter through N-terminal and C-terminal interactions with Nkx2.1 and Brg1, respectively. Therefore, Jmjd3 activates *SP-B* expression by acting as a bridge to promote the formation of Nkx2.1-Brg1 stable complexes.

In summary, through the generation of conventional and conditional *Jmjd3* KO mice, our study provides *in vivo* evidence that Jmjd3 is required for the early lung development, but dispensable for lung development at E9.5 or later embryonic stages. Furthermore, we have identified important Jmjd3 target genes that are critical for lung development and potential mechanisms by which Jmjd3 interacts with Brg1 and NKx2.1 to regulate histone methylation status in the promoter regions of these lung development genes.

## Materials and Methods

### Mice

Jmjd3 floxed mice (*Jmjd3^f/f^*) and *Jmjd3^−/−^* mice were generated by homologous recombination technique [17]. Mice were bred and maintained at the pathogen-free animal facilities of Baylor Medical School and the Houston Methodist Research Institute. Animal procedures were approved by the Animal Care and Use Committee at Baylor College of Medicine and Houston Methodist Research Institute. To generate inducible *Jmjd3* deletion in mice, TM (Sigma T5648) was dissolved in corn oil (Sigma) at a concentration of 10 mg/ml and intraperitoneally (IP) injected into 8-week-old mice at 9 mg TM/40 g of body weight [48], [49]. Pregnant mice received 3–4 mg TM/40 g of body weight by IP injection for 2 consecutive days. Pregnant mice were injected with TM at different time points to recover E4.5, E9.5, and E14.5 embryos. The newborn pups were genotyped with tail and lung tissue, and lung structure and development were assessed. The *Wnt1-Cre* strain was obtained from the Jackson Laboratory (022137). *SPC-Cre*, and *CCSP-Cre* and *Utx^floxed^* mice were kindly provided by Dr. Brigid Hogan (Duke University Medical Center), Dr. Frank DeMayo (Baylor College of Medicine) and Dr. Kai Ge (National Institutes of Health), respectively.

### RNA preparation, reverse transcription (RT)-PCR, and qPCR

Total RNAs were isolated from E17.5 lung tissues and primary MEFs using Trizol reagent (Invitrogen). A total of 1 µg of RNA was converted to cDNA using Superscript III Reverse Transcriptase (Qiagen) with random hexamer primers. qPCR was carried out using SYBR Green mix on the ABI 7000 PCR machine. *Jmjd3* expression in heterozygous and homozygous WT MEFs and WT and *Jmjd3* ^−/−^ E17.5 lung tissues was determined using the following Jmjd3 primer set: (forward) 5′-AAGTGGGGACAAGGAGACCT and (reverse) 5′-AAGTGGGGACAAGGAGACCT. WT and *Jmjd3* ^−/−^ E17.5 lung tissues were also evaluated for the expression of SP-A (5′-CTCCAGACCTGTGCCCATATG and 5′-ACCTCCAGTCATGGCACAGTAA), *SP-B* (5′-ACGTCCTCTGGAAGCCTTCA and 5′-TGTCTTCTTGGAGCCACAACAG), *SP-C* (5′-ACCCTGTGTGGAGAGCTACCA and 5′-TTTGCGGAGGGTCTTTCCT), *SP-D* (5′-CTCTGAGGCAGCAGATGGA and 5′-ATCAGGGAACAATGCAGCTT), *CC10* (5′-TCCTAACAAGTCCTCTGTGTAAGA and 5′-AGGAGACACAGGGCAGTGACA), *AQP-5* (5′-ATGAACCCAGCCCGATCTTT and 5′-ACGATCGGTCCTACCCAGAAG), *ABCA3* (5′-TTGCCCTCATTGGAGAGCCTG and 5′-TCCGGCCATCCTCAGTGGTGGG), *Nkx2.1* (5′-TCCAGCCTATCCCATCTGAACT and 5′-CAAGCGCATCTCACGTCTCA), *UTX* (5′-ATCCCAGCTCAGCAGAAGTT and 5′-GGAGGAAAGAAAGCATCACG), *Ezh2* (5′-GCCAGACTGGGAAGAAATCTG and 5′-TGTGCTGGAAAATCCAAGTCA), *Hoxa5* (5′-TCTCGTTGCCCTAATTCATCTTT and 5′-CATTCAGGACAAAGAGATGAACAGAA), *Hoxb1* (5′-TTGCCCTGGAAACTGTAAAG and 5′-AATTTGCCAACAACCCATC), *Esrrg* (5′- TGCACCGGGCTCTGTCAAGGAA and 5′-AATCCATGTGCGCCCGACAACC), *Reln* (5′-TCCAGGCTCAGCACCAAGCCAA and 5′- TGGATCTTGCCTTCTGACGCCCTT), *Kirrel3* (5′-AGAAAGTCACAGCTCCGCTCGGT and 5′-ACGGGAGGGTTGCAGAAAGGCT), *March4* (5′-GCCCCTCCCCTTGGTTCCATCAAA and 5′-CGAGGAGGAGAAAGCGAAGCCACT), *Fog2* (5′-CGCCTTTGTGGTGGACTTTGACT and 5′-GCTTCTCGTTGCCTCCCACTACA), *COUP-TFII* (5′-AGTACTGCCGCCTCAAAAAG and 5′-CGTTGGTCAGGGCAAACT), *Wt1* (5′-AATGCGCCCTACCTGCCCA and 5′- CCGTCGAAAGTGACCGTGCTGTAT) and *β-Actin* (5′-GTGGGCCGCTCTAGGCACCA and 5′-TGGCCTTAGGGTTCAGGG).

### Microarray

Microarray analysis was performed on RNA samples prepared from E17.5 *Jmjd3^+/+^* and *Jmjd3^−/−^* lungs using the Affymetrix Genechip microarray system. RNA quality assessment and microarray analysis were performed by the Microarray Core Facility of Baylor College of Medicine. RNA quality was checked using the NanoDrop ND-1000 spectrophotometer and Agilent 2100 Bioanalyzer. Total RNA samples of 250 ng were labeled using the 3′ IVT Express Kit with the new standard Affymetrix linear amplification protocol and then reverse transcribed to produce double-stranded cDNA. The cDNA product was used as a template for the *in vitro* transcription of biotin-labeled cRNA. The labeled cRNA was quantified using the NanoDrop ND-1000 spectrophotometer. A total of 15.0 µg of the labeled cRNA was fragmented and the concentration rechecked. A hybridization cocktail containing Affymetrix spike-in controls and fragmented labeled cRNA was loaded onto a GeneChip array. The array was hybridized for 16 h at 45°C with rotation at 60 rpm and then washed and stained with a strepavidin, R-phycoerythrin conjugate stain. Signal amplification was done using biotinylated anti-streptavidin. The stained array was scanned on the Affymetrix GeneChip Scanner 3000. The images were then analyzed and quality control metrics were recorded using Affymetrix GCOS software version 1.1.2. Pairwise comparisons were made between WT and *Jmjd3*-deficient lungs. Raw *P* values were adjusted by the Benjamini-Hochberg method for 5% false discovery rate to yield adjusted *p* values. The criteria for significance of differentially regulated genes were established as ≥1.3-fold change with an adjusted *P* value<0.05. Pathways were analyzed (Ingenuity Systems) to determine the ratios of known genes within each pathway that were significantly changed between WT and KO lungs.

### Immunoblotting analysis

Lung tissues were collected, ground into fine powders in liquid nitrogen, and transferred to lysis buffer (50 mM Tris-HCl [pH 7.4], 0.1% SDS) supplemented with protease inhibitor cocktail (cOmplete Mini Protease Inhibitor, 14132300) and 2 µM phenylmethylsulphonyl fluoride (PMSF). Cells were washed in phosphate-buffered saline (PBS), harvested, and lysed in RIPA buffer supplemented with protease inhibitor cocktail and 2 µM PMSF. After sonication and centrifugation, protein supernatants were collected, and protein concentrations were determined using a Bradford Protein Assay Kit (Sigma). Protein extracts were boiled in SDS sample buffer for 5 min, loaded directly onto a 4–12% SDS gel, transferred onto nitrocellulose membranes (Bio-Rad), blocked with 5% milk, and incubated with primary and corresponding secondary antibodies. The following antibodies were used: anti-H3K4me1 (07-436), anti-H3K4me2 (07-030), anti-H3K4me3 (07-473), anti-H3K9me1 (07-450), anti-H3K9me2 (07-441), anti-H3K9me3 (07-442), anti-H3K27me1 (07-448), anti-H3K27me2 (07-452), and anti-H3K27me3 (07-449) from Millipore; anti-SP-A (sc-7700), anti-AQP-5 (sc-9891); anti-SP-B (sc-13978), anti-SP-C (sc-13979), anti-SP-D (sc-13980), and anti-CC10 (sc-25555) from Santa Cruz Biotechnology; anti-H3(9715) and Ezh2 (4905) from Cell Signaling; anti-CD11b (17-0113-81), anti-F4/80 (15-4801-82), and anti-Gr1 (17-5931-81) from eBioscience; mouse anti-Flag, anti-HRP-flag, and anti-β-actin from Sigma; rabbit anti-UTX (ab36938) from (Abcam); and anti-Jmjd3 (ab1022a) from Abgent.

### Immunohistochemistry and tissue staining

Tissues and embryos were fixed in 10% neutral formalin. The fixation time was dependent on embryonic stage: E<12.5 were fixed for 2 h, E12.5-E17 for 4 h, and E>17.5 for 24–48 h as previously described [50]. After fixation, tissues and embryos were embedded in paraffin and cut into 5-µm thick sections. H&E staining and immunostaining were performed according to standard protocols [51]. MEFs were isolated from WT and *Jmjd3* KO embryos at E11.5 [51]. Immunocytofluorescence was performed according to a standard protocol using primary antibodies as mentioned above and corresponding goat anti-FITC-conjugated rabbit secondary antibodies (115-096-003; Jackson ImmunoResearch Laboratories) or goat anti-Texas Red-conjugated mouse secondary antibodies (81–6114; ZyMax Grade).

### ChIP-PCR and ChIP-qPCR analyses

Chromatin was prepared from lung tissues of E17.5 embryos. ChIP-PCR and ChIP-qPCR were performed using Jmjd3 antibody (Abgent) and selected histone marks, respectively. Immunoprecipitated chromatin DNA and input were used for ChIP-PCR and ChIP-qPCR with specific primers. Briefly, whole lungs of E17.5 embryos were quickly chopped into tiny pieces with a scalpel and fixed in 1% formaldehyde in 1X PBS at room temperature for 15 min. Glycine was added to a final concentration of 0.125 M. Samples were rotated at room temperature for another 5 min, centrifuged at low speed for 5 min, and the supernatants decanted. The pellets were washed once with ice-cold 1X PBS and disaggregated mechanically through a 0.5-µm cell strainer in 1 ml of ice-cold 1X PBS. Immunoprecipitation was performed on single cell suspensions using the ChIP Assay Kit according to the manufacturer's instructions (Millipore, 17–295). Antibodies used included anti-Jmjd3; anti-mono-, di-, and trimethylated H3K4 antibodies; anti-mono-, di-, and trimethylated H3K9 antibodies; and anti-mono-, di-, and trimethylated H3K27 antibodies. DNA binding of Jmjd3 and methylation of lung marker genes including *AQP-5*, *SP-A*, *SP-B*, *SP-C*, *SP-D*, and *CC10* were evaluated by ChIP-PCR and ChIP-qPCR using the following specific primers: AQP-5-AF, 5′-AACCTGCGGAGGGGGAAGGT; AQP-5-AR, 5′-CGTCCCCACCCCCACTCCAT; AQP-5-BF, 5′-CACCACCCCAGGGTCCCCAA; AQP-5-BR, 5′-CCTGCTCTGCGCTCGGCAAT; AQP-5-CF, 5′-CAGGAGAGCCCAGCACGCAC; AQP-5-CR, 5′-CAGTGTAGACTGGCCCGGCG, SP-A-AF, 5′-GCACACGTACGGAAGCCGGA; SP-A-AR, 5′-CCTGCGGTGCTCAGTGGCAA; SP-A-BF, 5′-CCTTTCTGCTTCTTTCCTATGGCCG; SP-A-BR, 5′-AGCAAAACATCAGAACAACCAAAACTA; SP-A-CF, 5′-ATGGCTGCTTCCTGTCCGGC; SP-A-CR, 5′-CCCGCACACAGAGCCTGCAA, SP-B-AF, 5′-AGGGCCCGGACACATAGAGG; SP-B-AR, 5′-CTGAGGCCCAGGGCAGAGGT; SP-B-BF, 5′-GGGTGTGAGGTGACACGCCG; SP-B-BR, 5′-CTGTGGTGGGGGTGACCACG; SP-B-CF, 5′-TGTCACCAGTGGCACAGTGGAA; SP-B-CR, 5′-AGGTGAGCACTGCCATACCAGG; SP-B-DF: 5′-GTAGAGGATTGAGAAGCCT; SP-B-DR, 5′-CAGCCTGACTTTGTTCAC; SP-B-EF, 5′-CGTGACTCTCTGAAGAAGGC; SP-B-ER: 5′-AAACGACACCCTGGAAGTG; SP-B-FF, 5′-CCCTTGTTTGACGGTGAA; SP-B-FR: 5′-TGGCTGCCTACTGCTTAGT; SP-C-AF, 5′-ACAGGCAATCCCAGATCGCTGA; SP-C-AR: 5′-GTCCCAGCCATCTCTGCCCCT; SP-C-BF, 5′-AGAGGGGCAGAGATGGCTGGG; SP-C-BR, 5′-GCTTGGGACAGCACCTGGGG; SP-C-CF, 5′-AGGCCCAGTCCTTCACCCCTG; SP-C-CR, 5′-GCCTACTGGAGGATGGACAGTCA; SP-D-AF, 5′-TCAGAGGACTAATGACAGCCTT; SP-D-AR, 5′-CAGCAGGGACAGACATACCA; SP-D-BF, 5′-GGAAGATGGAAGAACAAGGG; SP-D-BR, 5′-CTACAAAGGCAGCAACCTG; SP-D-CF, 5′-TGTGTGTGTGTGTGTGTGTGA; SP-D-CR, 5′-CACCTCTGTTTGTCAGGCTC; CC10-AF, 5′-TCCCACCAGCACCATAGTA; CC10-AR, 5′-CACCTTCTCCATTTCCACA; CC10-BF, 5′-CTAACAATGCCCAAGAATCG ; CC10-BR, 5′-GGAGACCCTTCAGGAATCA; CC10-CF, 5′-CTCCACTGCCTGAATACTCC; CC10-CR, 5′-ACTTGGTCATCTTCTCCGTG.

### ChIP-Seq analysis

Lungs of E17.5 *Jmjd3^+/+^* and *Jmjd3^−/−^* embryos were collected and prepared for ChIP-PCR as previously described [17]. A total of 200 ng of DNA was used for the ChIP-Seq library construction. Illumina sequencing was performed as previously described [52]–[54]. Sequencing reads from H3K4me3 and H3K27me3 ChIP-Seq libraries were aligned to the mouse mm8 genome using ELAND software. To reduce PCR amplification bias, only one uniquely mapped read per genomic position was retained. The histone modification changes upon *Jmjd3* knockout were assessed using the fold changes of background-subtracted read densities around 5 kb and 2 kb of the TSSs. The statistical significance of the fold change was assessed using the MA-plot-based method. The fold change and the false discovery rate cutoffs were set to 1.5 and 10%, respectively.

### Statistical analysis

Data are represented as mean ± standard deviation (SD) where indicated. Statistical analysis was performed using the Student's *t* test with GraphPad Prism 4.0 software. A *P* value<0.05 was considered significant.

## Supporting Information

Figure S1 Jmjd3 deficiency induces developmental defects in the lung. (**A**) H&E staining of *Jmjd3*-deficient lung tissues at P0. Type I and type II cells and capillary beds were altered in *Jmjd3*-deficient lungs. Bar = 50 µm. (**B**) All lobes were smaller in *Jmjd3*-deficient lungs at E17.5 compared with WT lungs. L, left lobe; S, superior lobe; P, post-caval lobe; M, middle lobe; I, inferior lobe. (**C**) H&E staining of *Jmjd3*-deficient lung tissues at E17.5.
(TIF)

Figure S2 UTX does not compensate for Jmjd3 deficiency. All *Jmjd3^f/f^*:*Utx^f/f^*:*SPC-Cre*, *Jmjd3^f/f^:SPC-Cre*, and *Utx^f/f^:SPC-Cre* pups at P0 appeared to be normal and were able to grow to adulthood.
(TIF)

Figure S3 Gene expression in *Jmjd3* KO tissues. (**A**) Pre-Bötzinger complex-specific gene expression in the brainstem of *Jmjd3* KO and WT mice at E17.5. and P0. (**B**) Pre-Bötzinger complex-specific gene expression in *Jmjd3* KO and WT E17.5 lung tissues. (**C**) Respiratory muscle gene expression in diaphragm and intercostal muscle at P0. Data are presented as the mean ± SD from three independent experiments. *P<0.05, **P<0.01 (Student's *t* test).
(TIF)

Figure S4 Jmjd3 deletion affects global H3 histone methylation and lung marker gene promoter methylation. (**A**) Total methylation of H3K27, H3K4, and H3K9 in *Jmjd3*-deficient lungs. (**B**) Immunostaining of WT and *Jmjd3* ^−/−^ primary MEFs with anti-mono-, anti-di-, and anti-trimethylated H3K4, H3K9, and H3K27 antibodies. DAPI nuclear staining is shown in blue. Texas-red (TR) staining indicates histone methylation. (**C**) Ectopic expression of *Jmjd*3 rescues total H3K27me3 in *Jmjd3* ^−/−^ MEFs. Lane 1, wild-type; lane 2, *Jmjd3* ^−/−^ MEFs; lane 3, *Jmjd3* ^−/−^ MEFs infected with control retrovirus; lane 4, *Jmjd3* ^−/−^ MEFs infected with retroviruses expressing *Flag-Jmjd3*. (**D**) Immunostaining of H3K27 methylation in *Jmjd3* ^−/−^ MEFs infected with *Flag-Jmjd3* (*F-Jmjd3*) retrovirus. White arrowheads indicate infected cells.
(TIF)

Figure S5 ChIP-Seq and ChIP-PCR analysis of lung marker and RRG genes. (**A**) ChIP-Seq analysis of H3K4me3 and H3K27me3 methylation in the regulatory and gene body regions of genes related to lung development. Scale = 2 kb. (**B**) ChIP-Seq analysis of H3K27me3 and H3K4me3 levels in several RRG-related genes in *Jmjd3* KO and WT E17.5 lung tissues. Open boxes indicate 5 kb regions around TSS sites. Red arrows indicate the transcription direction. (**C**) ChIP-PCR analysis of Jmjd3 binding to *AQP-5*, *SP-A*, *SP-C*, *SP-D*, and *CC10* proximal promoter regions. The lung tissues of *Jmjd3* ^+/+^ and *Jmjd3* ^−/−^ embryos at E17.5 were formalin fixed, homogenized, and sonicated before immunoprecipitation with anti-Jmjd3 antibody. The binding regions were analyzed by PCR with specific primers. The designed primers and their location on the gene promoters (*right panel*). IgG was used as negative control. The red ovals represent Jmjd3 binding to the indicated regions. (**D**) ChIP-qPCR analysis of H3K27 and H3K4 mono-, di-, and trimethylation levels in the regions upstream of the TSS sites of *AQP-5*, *SP-D*, and *CC10* in *Jmjd3* ^−/−^ and WT lungs. The location of the designed primers A, B, and C is around −1000 bp, −500 bp, and +1 bp, respectively (*top panel*). Data are presented as the mean ± SD of methylation enrichment (fold) from three independent experiments. *P<0.05 (Student's *t* test).
(TIF)

Figure S6 UTX does not interact with Nkx2.1. Immunoprecipitation and immunoblot analysis of 293T cells expressing HA-tagged *Utx* and FLAG-tagged *Nkx2.1* do not show an interaction between UTX and Nkx2.1.
(TIF)

Figure S7 Catalytic domain of Jmjd3 is required for enhancing SP-B-promoter-driven luciferase activity. Up, the measurement of luciferase activity; bottom, protein expression level of Jmjd3 fragments, FLAG-Brg1, FLAG-Nkx2.1, and β-actin. NS, non-specific bands.
(TIF)

Table S1 Genotyping analysis of embryos and newborns from *Jmjd3^+/−^* breeding pairs.
(JPG)

Table S2 Microarray analysis of gene expression in *Jmjd3^−/−^* and WT embryonic lungs at E17.5.
(XLSX)

Table S3 ChIP-Seq analysis of H3K4 and H3K27 methylation around +/− 5 kb of TSS.
(XLSX)

Table S4 ChIP-Seq analysis of H3K4 and H3K27 methylation around −2 kb of TSS.
(XLSX)
