# Correction: Stage-Dependent and Locus-Specific Role of Histone Demethylase Jumonji D3 (JMJD3) in the Embryonic Stages of Lung Development

**DOI:** 10.1371/journal.pgen.1010701

**Published:** 2023-03-30

**Authors:** Qingtian Li, Helen Y. Wang, Iouri Chepelev, Qingyuan Zhu, Gang Wei, Keji Zhao, Rong-Fu Wang

Figure panels Fig 3D and Fig 6H contain duplicated images. The authors have provided corrected versions of Fig 3 and Fig 6 here.

**Fig 3:**
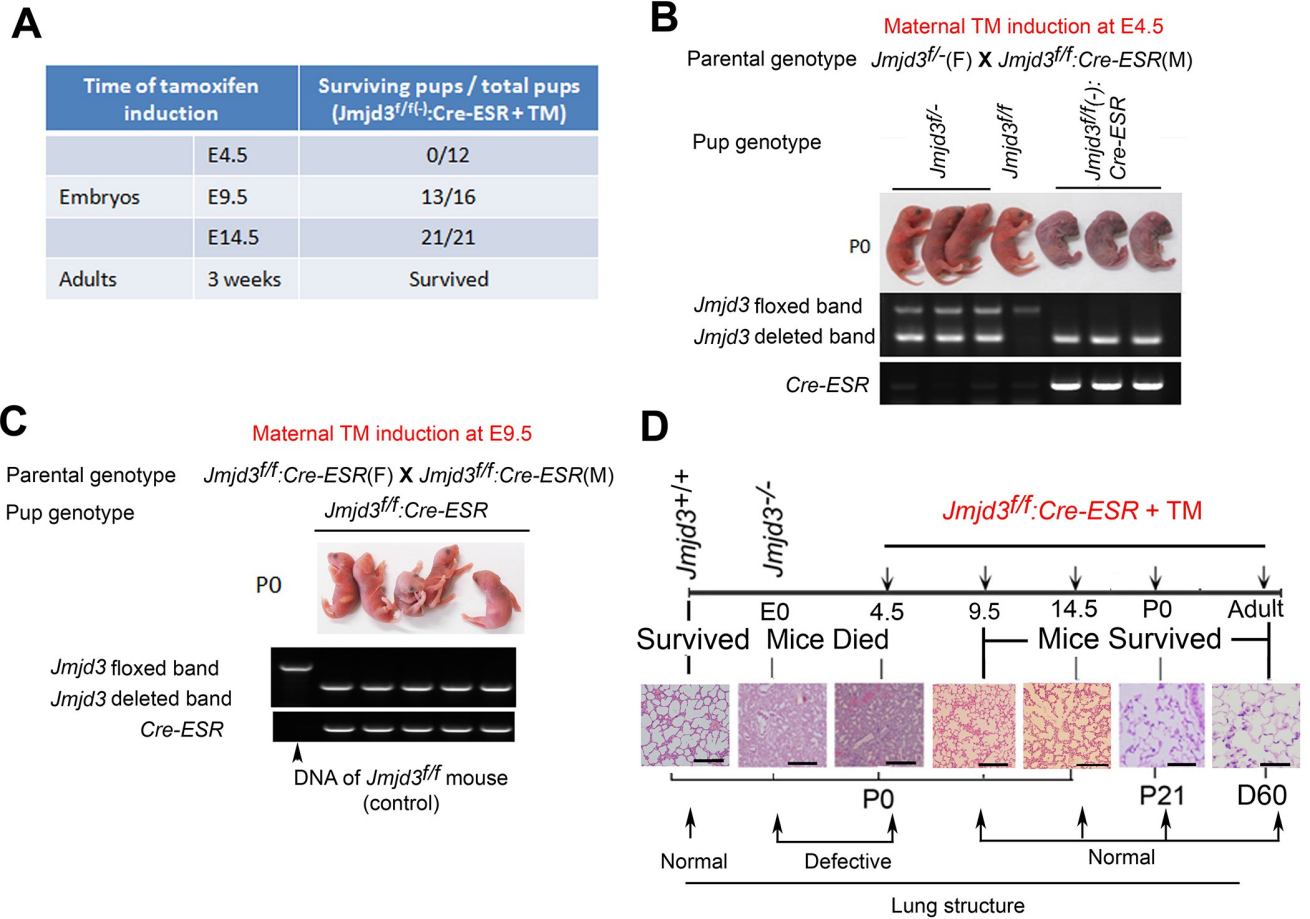
Stage-specific deletion reveals that Jmjd3 is dispensable for lung development in later E9.5 embryonic stage. (A) Jmjd3f/f mice were crossed with CAG-Cre/ESR mice in which Cre expression is globally induced with TM. The survival of Jmjd3f/f:Cre/ESR pups from maternal mice treated with TM at E4.5, E9.5, and E14.5 was determined. (B) PCR analysis of Jmjd3 deletion efficiency in Jmjd3f/f:Cre/ESR pups from maternal mice treated with TM at E4.5. (C) PCR analysis of Jmjd3 deletion efficiency in Jmjd3f/f:Cre/ESR pups from maternal mice treated with TM at E9.5. (D) H&E staining showing the stage-dependent effects of Jmjd3 deletion on lung architecture and its correlation with embryo viability. Bar  =  500 µm.

**Fig 6:**
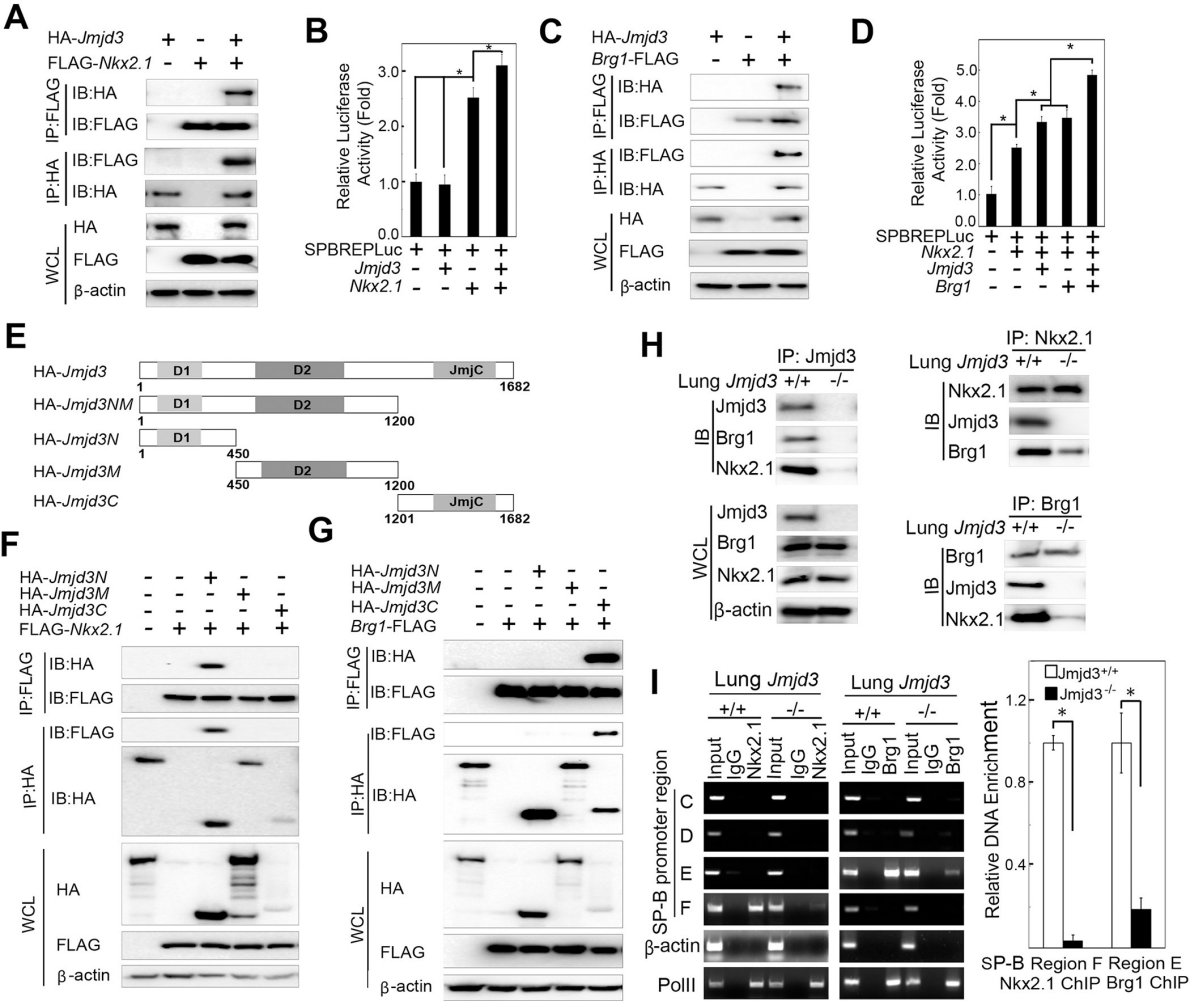
Jmjd3 coregulates SP-B expression with Nkx2.1 and Brg1. (A) The interaction between Jmjd3 and Nkx2.1 was evaluated by coimmunoprecipitation (CoIP) analysis of 293T cells expressing HA-tagged Jmjd3 and FLAG-tagged Nkx2.1. (B) A cell-based luciferase assay was used to evaluate SP-B promoter activity in 293T cells cotransfected with Nkx2.1, Jmjd3, and mouse SP-B promoter-linked episomal luciferase vector (containing Nkx2.1 binding sites). (C) The interaction between Jmjd3 and Brg1 was determined by CoIP analysis of 293T cells expressing HA-tagged Jmjd3 and FLAG-tagged Brg1. (D) Cell-based luciferase assay was used to evaluate SP-B promoter activity in 293T cells cotransfected with Nkx2.1, Jmjd3, Brg1, and mouse SP-B promoter-linked episomal luciferase vector. *P<0.05 (Student’s t test). (E) Generation of Jmjd3 deletion constructs. Numbers represent the corresponding amino acid residue position in the Jmjd3 coding region. (F) CoIP analysis of the interaction between Nkx2.1 and HA-tagged truncated Jmjd3 proteins. (G) CoIP analysis of the interaction between Brg1 and truncated Jmjd3 proteins. (H) CoIP analysis of the interaction among endogenous Nkx2.1, Brg1, and Jmjd3 in WT and Jmjd3-deficient lung tissues. (I) ChIP-PCR and ChIP-qPCR analysis of Nkx2.1 and Brg1 binding to SP-B promoter regions in WT and Jmjd3-deficient lung tissues. *P<0.05 (Student’s t test).
