# Supplementary figures and images for: Stage-Dependent and Locus-Specific Role of Histone Demethylase Jumonji D3 (JMJD3) in the Embryonic Stages of Lung Development
Source: PLoS Genet. 2014 Jul 31;10(7):e1004524. doi: 10.1371/journal.pgen.1004524 (PMC4117460; doi:10.1371/journal.pgen.1004524)

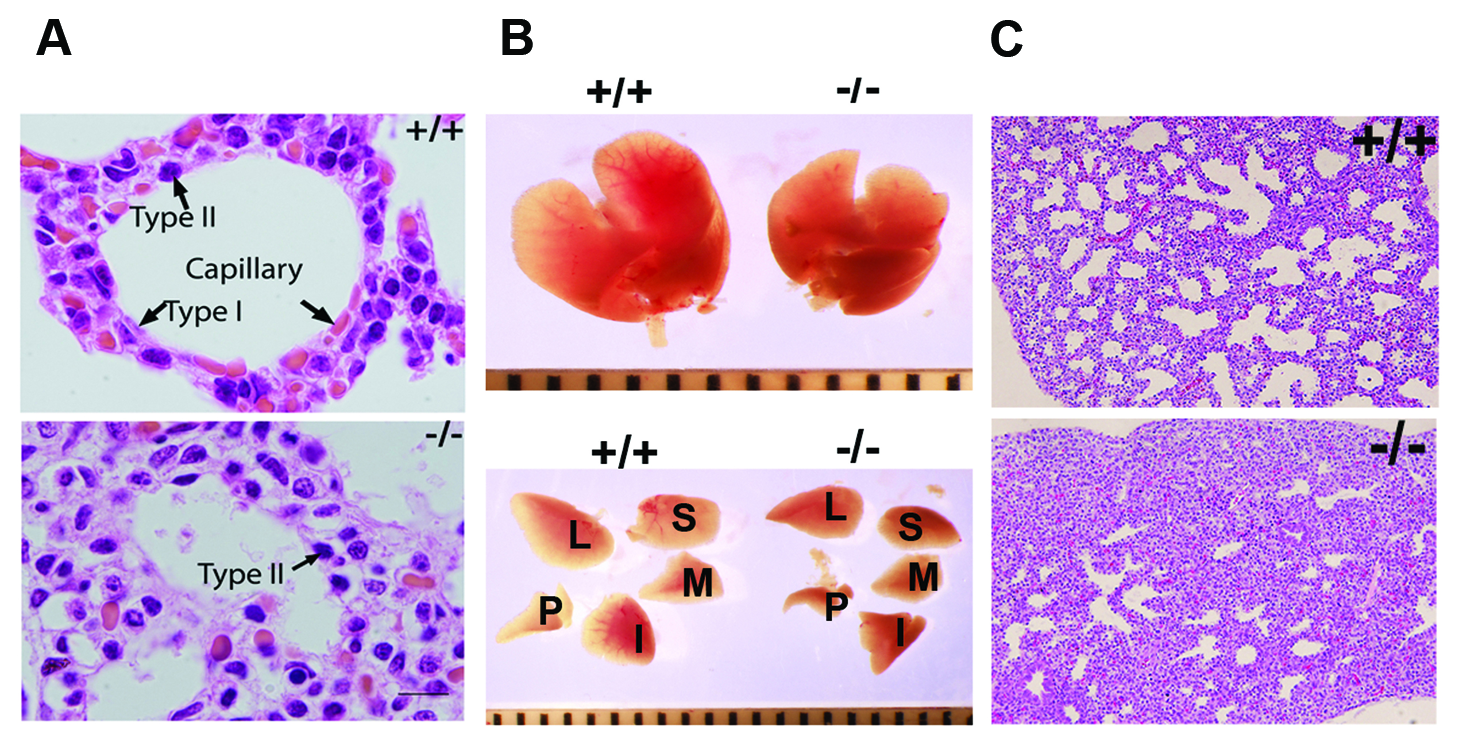

Supplement: Figure S1 — Jmjd3 deficiency induces developmental defects in the lung. (A) H&E staining of Jmjd3-deficient lung tissues at P0. Type I and type II cells and capillary beds were altered in Jmjd3-deficient lungs. Bar = 50 µm. (B) All lobes were smaller in Jmjd3-deficient lungs at E17.5 compared with WT lungs. L, left lobe; S, superior lobe; P, post-caval lobe; M, middle lobe; I, inferior lobe. (C) H&E staining of Jmjd3-deficient lung tissues at E17.5. (TIF) [file pgen.1004524.s001.tif]

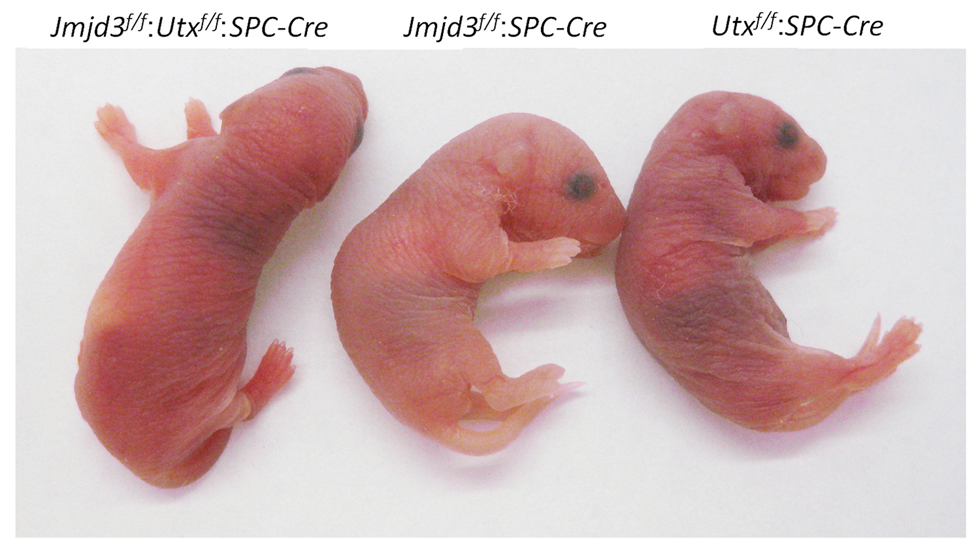

Supplement: Figure S2 — UTX does not compensate for Jmjd3 deficiency. All Jmjd3f/f:Utxf/f:SPC-Cre, Jmjd3f/f:SPC-Cre, and Utxf/f:SPC-Cre pups at P0 appeared to be normal and were able to grow to adulthood. (TIF) [file pgen.1004524.s002.tif]

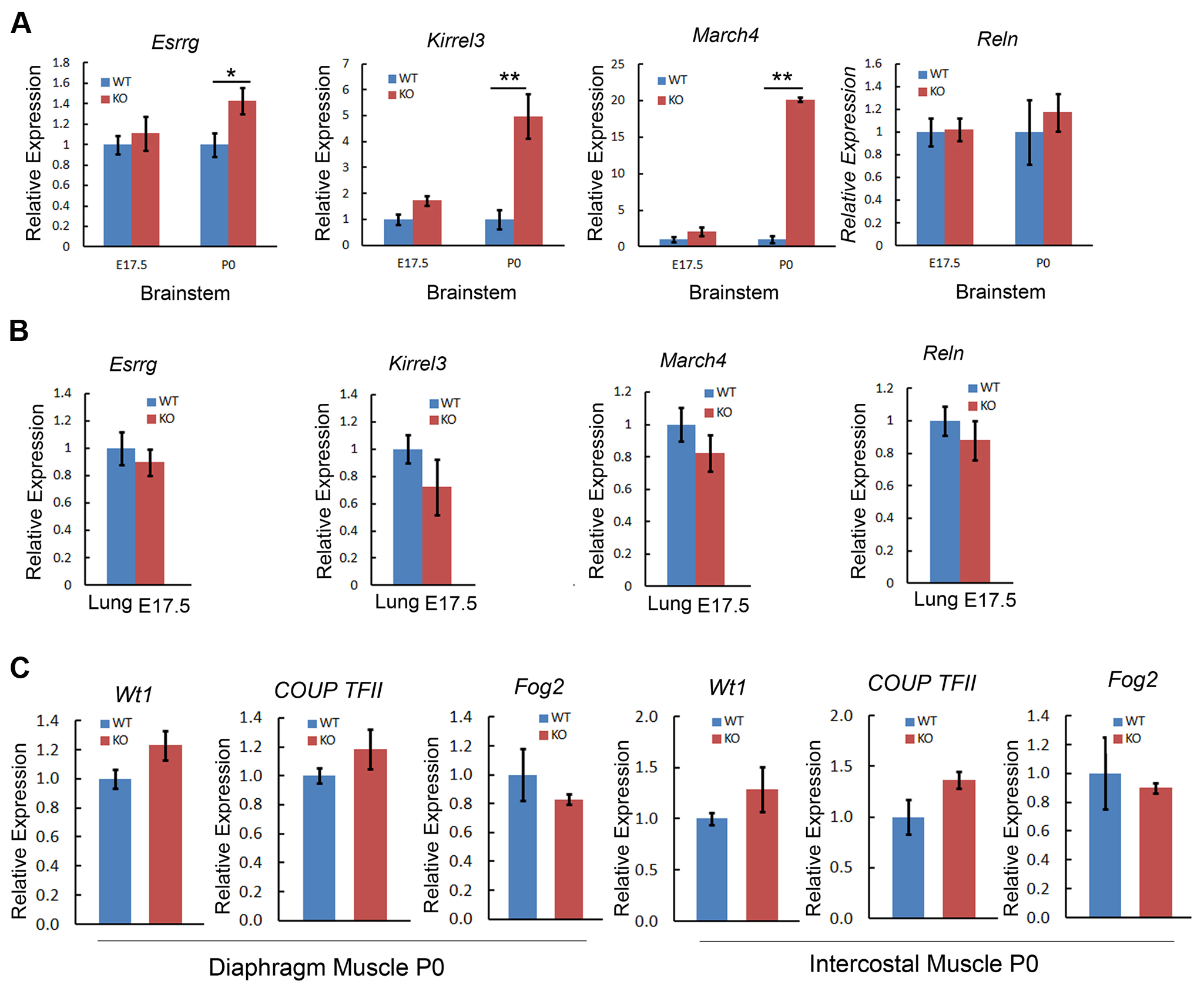

Supplement: Figure S3 — Gene expression in Jmjd3 KO tissues. (A) Pre-Bötzinger complex-specific gene expression in the brainstem of Jmjd3 KO and WT mice at E17.5. and P0. (B) Pre-Bötzinger complex-specific gene expression in Jmjd3 KO and WT E17.5 lung tissues. (C) Respiratory muscle gene expression in diaphragm and intercostal muscle at P0. Data are presented as the mean ± SD from three independent experiments. *P<0.05, **P<0.01 (Student's t test). (TIF) [file pgen.1004524.s003.tif]

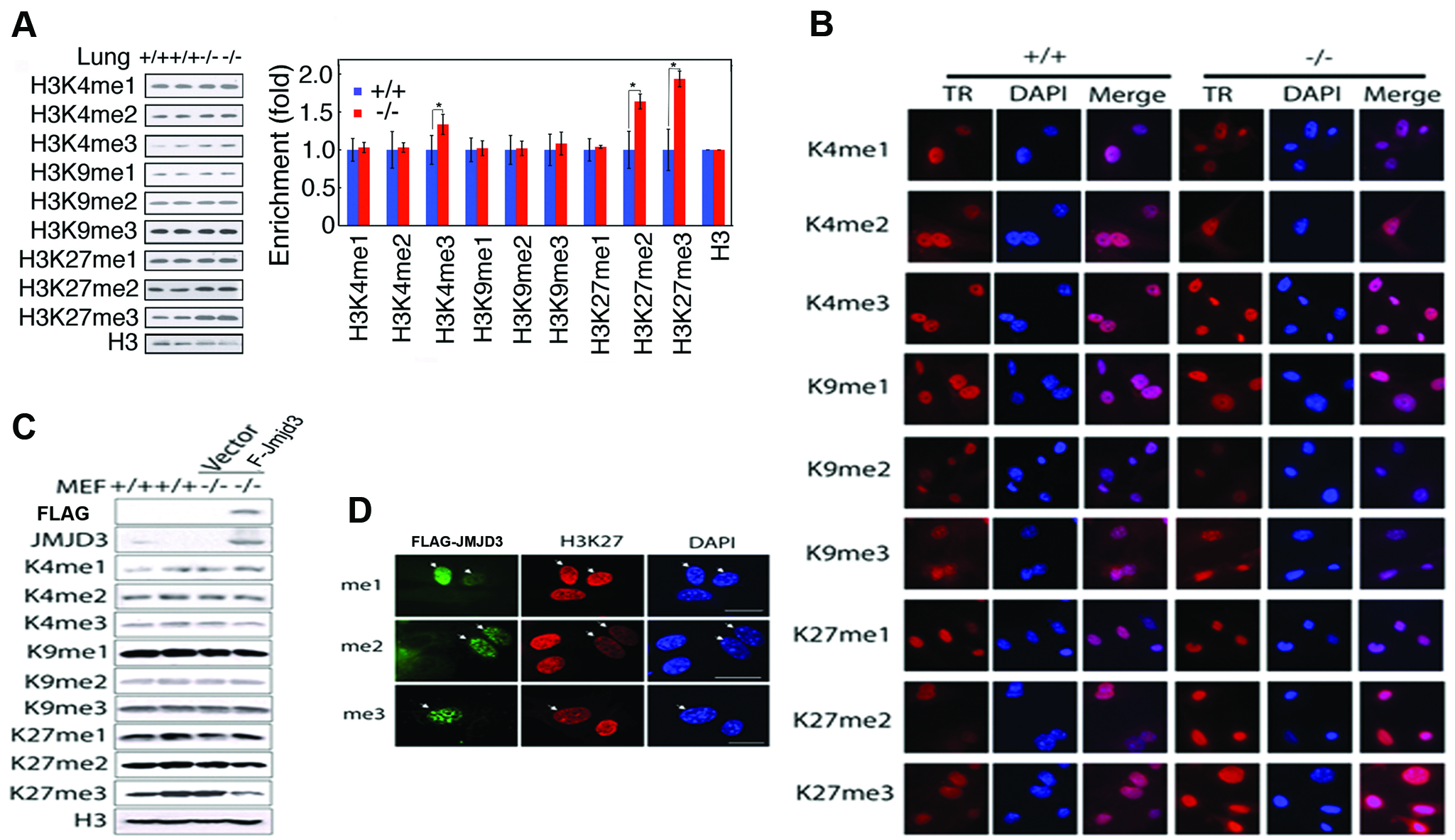

Supplement: Figure S4 — Jmjd3 deletion affects global H3 histone methylation and lung marker gene promoter methylation. (A) Total methylation of H3K27, H3K4, and H3K9 in Jmjd3-deficient lungs. (B) Immunostaining of WT and Jmjd3 −/− primary MEFs with anti-mono-, anti-di-, and anti-trimethylated H3K4, H3K9, and H3K27 antibodies. DAPI nuclear staining is shown in blue. Texas-red (TR) staining indicates histone methylation. (C) Ectopic expression of Jmjd3 rescues total H3K27me3 in Jmjd3 −/− MEFs. Lane 1, wild-type; lane 2, Jmjd3 −/− MEFs; lane 3, Jmjd3 −/− MEFs infected with control retrovirus; lane 4, Jmjd3 −/− MEFs infected with retroviruses expressing Flag-Jmjd3. (D) Immunostaining of H3K27 methylation in Jmjd3 −/− MEFs infected with Flag-Jmjd3 (F-Jmjd3) retrovirus. White arrowheads indicate infected cells. (TIF) [file pgen.1004524.s004.tif]

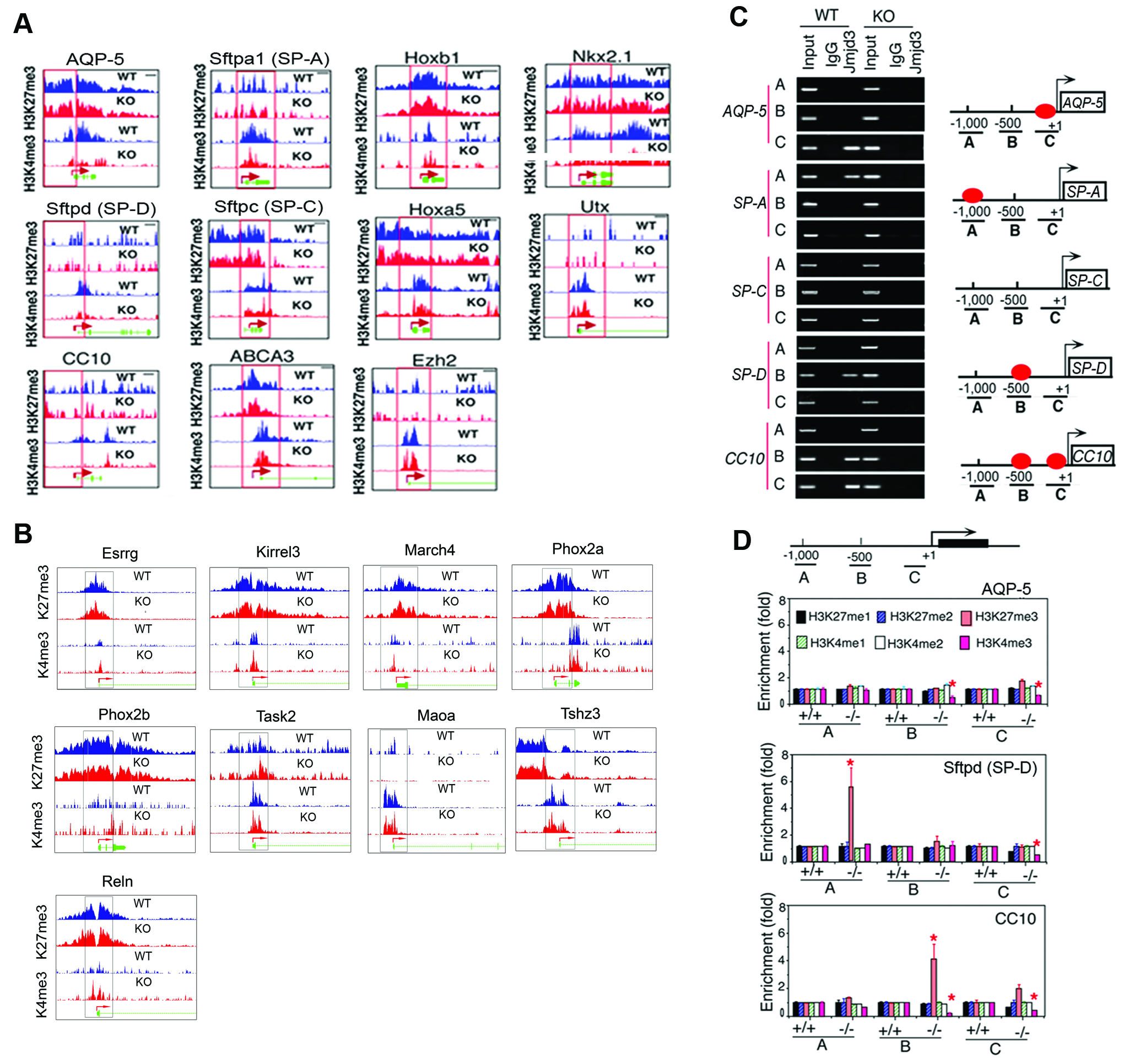

Supplement: Figure S5 — ChIP-Seq and ChIP-PCR analysis of lung marker and RRG genes. (A) ChIP-Seq analysis of H3K4me3 and H3K27me3 methylation in the regulatory and gene body regions of genes related to lung development. Scale = 2 kb. (B) ChIP-Seq analysis of H3K27me3 and H3K4me3 levels in several RRG-related genes in Jmjd3 KO and WT E17.5 lung tissues. Open boxes indicate 5 kb regions around TSS sites. Red arrows indicate the transcription direction. (C) ChIP-PCR analysis of Jmjd3 binding to AQP-5, SP-A, SP-C, SP-D, and CC10 proximal promoter regions. The lung tissues of Jmjd3 +/+ and Jmjd3 −/− embryos at E17.5 were formalin fixed, homogenized, and sonicated before immunoprecipitation with anti-Jmjd3 antibody. The binding regions were analyzed by PCR with specific primers. The designed primers and their location on the gene promoters (right panel). IgG was used as negative control. The red ovals represent Jmjd3 binding to the indicated regions. (D) ChIP-qPCR analysis of H3K27 and H3K4 mono-, di-, and trimethylation levels in the regions upstream of the TSS sites of AQP-5, SP-D, and CC10 in Jmjd3 −/− and WT lungs. The location of the designed primers A, B, and C is around −1000 bp, −500 bp, and +1 bp, respectively (top panel). Data are presented as the mean ± SD of methylation enrichment (fold) from three independent experiments. *P<0.05 (Student's t test). (TIF) [file pgen.1004524.s005.tif]

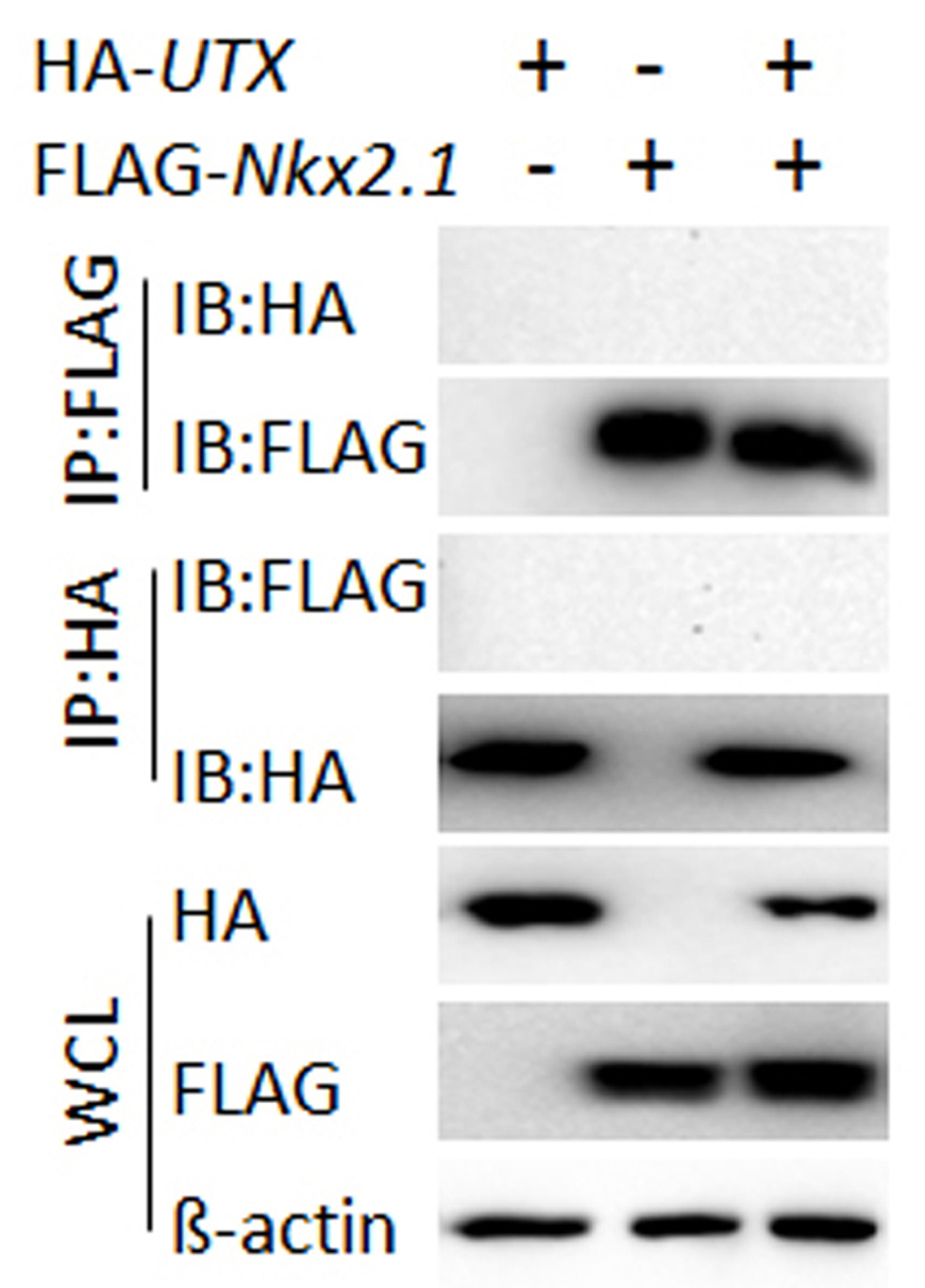

Supplement: Figure S6 — UTX does not interact with Nkx2.1. Immunoprecipitation and immunoblot analysis of 293T cells expressing HA-tagged Utx and FLAG-tagged Nkx2.1 do not show an interaction between UTX and Nkx2.1. (TIF) [file pgen.1004524.s006.tif]

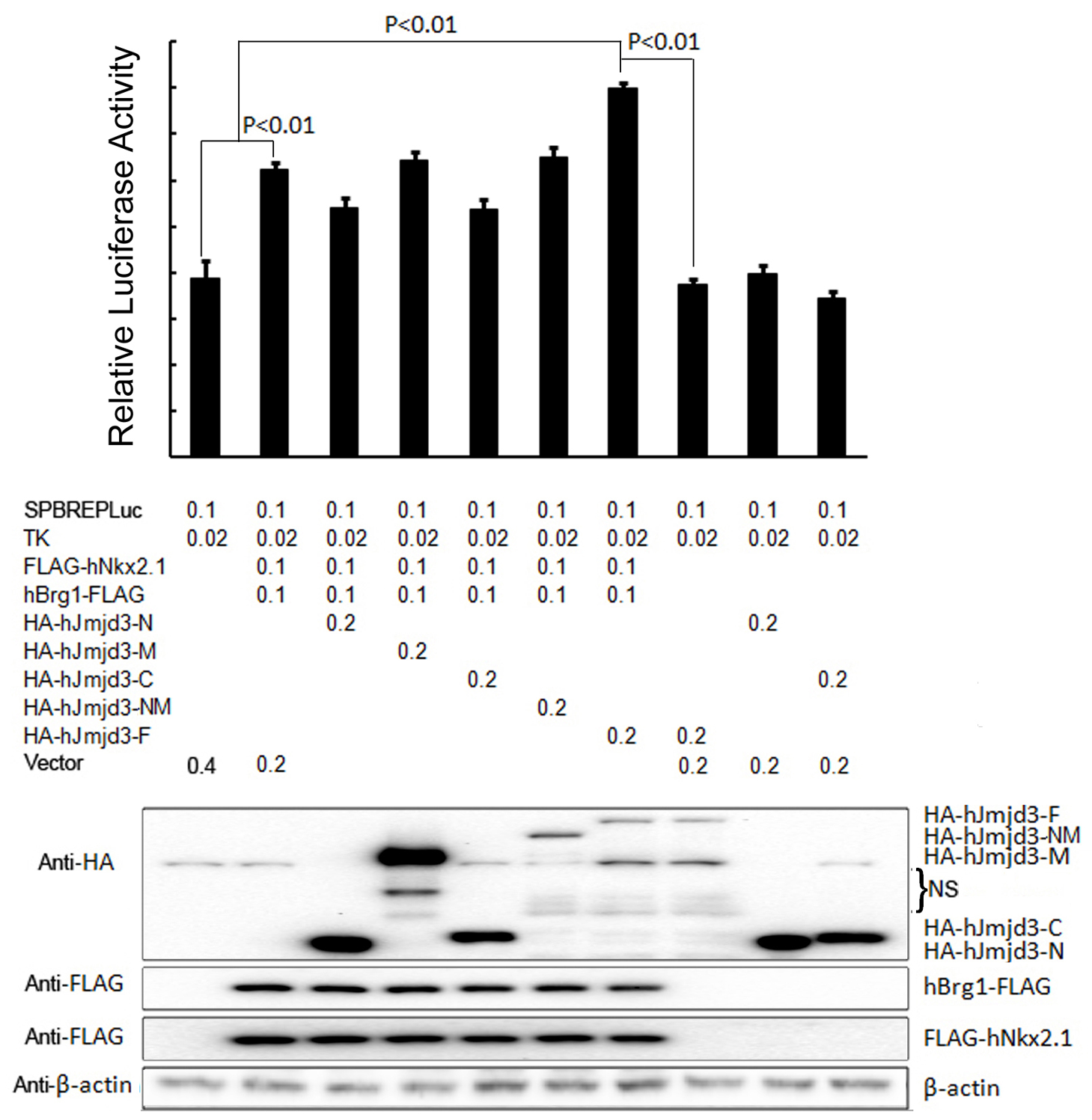

Supplement: Figure S7 — Catalytic domain of Jmjd3 is required for enhancing SP-B-promoter-driven luciferase activity. Up, the measurement of luciferase activity; bottom, protein expression level of Jmjd3 fragments, FLAG-Brg1, FLAG-Nkx2.1, and β-actin. NS, non-specific bands. (TIF) [file pgen.1004524.s007.tif]

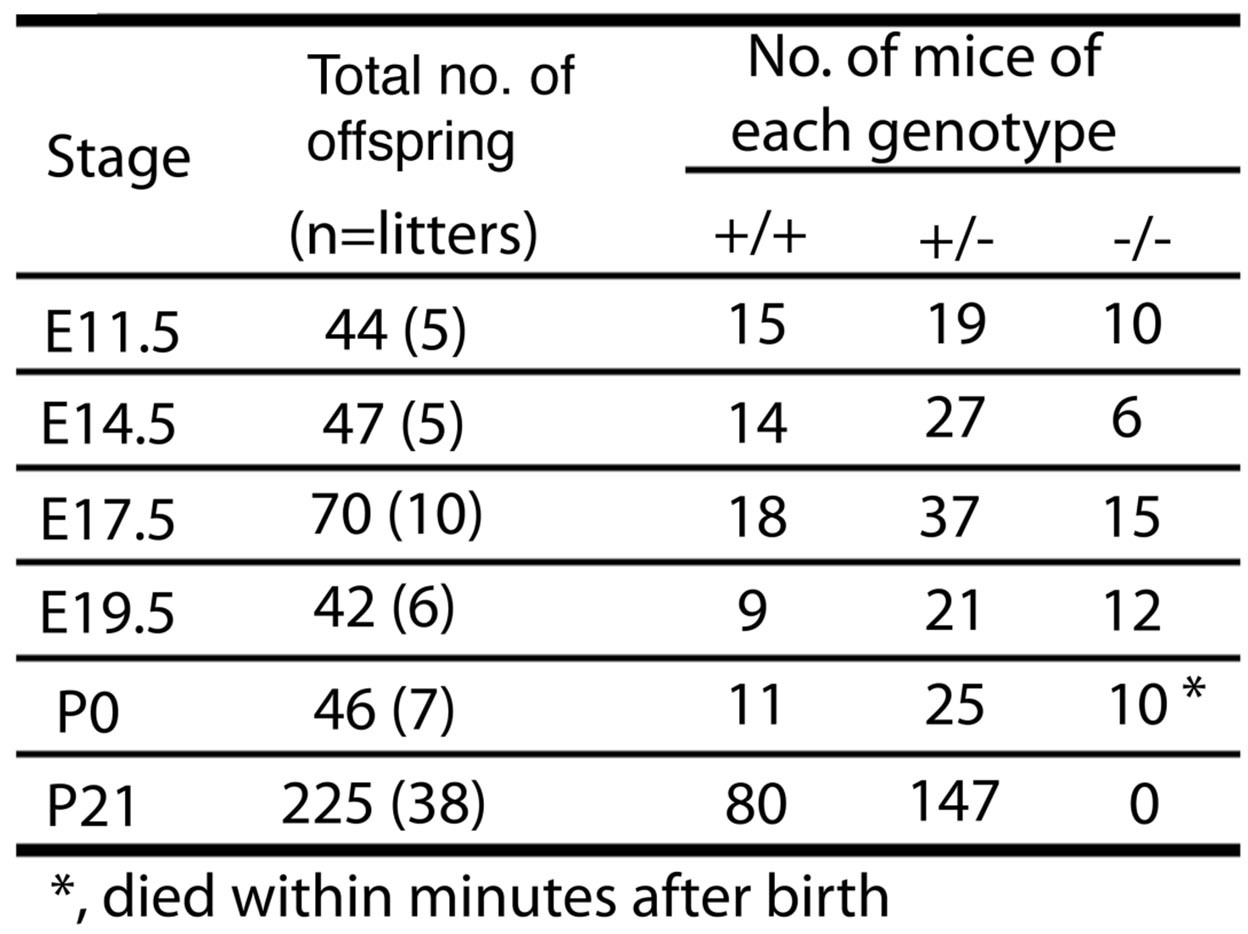

Supplement: Table S1 — Genotyping analysis of embryos and newborns from Jmjd3+/− breeding pairs. (JPG) [file pgen.1004524.s008.jpg]
